# Supplementary material for: Isolation and characterization of a novel bacteriophage WO from Allonemobius socius crickets in Missouri
Source: PLoS One. 2021 Jul 1;16(7):e0250051. doi: 10.1371/journal.pone.0250051 (PMC8248633; doi:10.1371/journal.pone.0250051)
Supplement: S1 File — Sequences in FASTA format of the assembled scaffolds 1–4, the head decoration protein, major capsid protein, and minor capsid protein. (DOCX) [file pone.0250051.s001.docx]

>Phage wAsoc Assembly scaffold 1

CCAGGGAAATGGAGAACGGAAAGAACTCCTTATTTAAAAGAAATCATGGATTCACTTTCTCCGTCCTCACCAGCAGAAAAAGTAGTATTCATGAAAGGAGCGCAGATTGGGGGAACAGAAGCTGGTAACAATTGGATTGGCTATATTATCGATCAAACACCAGGTCCAATGCTAGTAGTACAGCCAACAGTTGAAATGGGAAAACGTTGGTCAAAAGGAAGATTTGCGCCATTAATAGAGAGTACACCATGTTTAAAAAATAAAGTAAAAGATCCAAGGTCAAGAGACTCAGGCAATACTGTACAAAGTAAGGAATTTCCAGGTGGAATAGTAGTAATAACCGGAGCAAATAGCAGTGTAGGACTTCGTTCTATGCCAGTAAAATATCTCTTTCTTGATGAGATAGATGCATATCCAGGAGATTCAGGCGGAGAAGGAGATCCAGTGCTGCTCAGTATTGCTCGAACTAATACTTTTGCGCGGCGAAAGATTTTTTTAGTGTCAACACCAACGATTCATGGAATAAGCAGAATTGAGAAGGAATTTGAAGCAACAGATAAGAGATATTTTTTTGTACCATGTCCACATTGTAATTACTATCAAGTTCTGAAATGGTCACAAATAAAATGGGAAAACAACGACTCAAGAACAGCACATTATGTCTGCACTGAATGTAGCGGCAAGATAGAAAATCATCAAAAGACAGAGATGCTTGAACGTGGAGAATGGAGGCCTACTAATAGAGTAAAAGGTGAGAAAAAAGGATTTCATCTTTCAAGTCTTTATAGTCCAGTGGGCTGGTATAGCTGGACTCAAGCAGTAGAAGATTTTCTCCATGCCAAAGAAAGTGAACAATTACTGAAAGTTTGGATAAATACTACGCTTGGAGAAACTTGGGTAGACAAAGGAGAAGTACCAGACTGGAAGCAATTATTTAACAGGAGAGAATTTTTTCCCATAGGCACAGTACCAAAAGGCGAAGTGGTACTCACAGCAGGTGTTGATGTCCAAAAAGATCGTTTAGAAGTAGAAGTTGTAGCATGGGGAAAAAGCCGCGAAAGTTGGTCAATAGACTATCGAGTATTTGAAGGAGATACAGGAGGTGGAGAAGTATGGGGAAAACTCTCTGAGCTCTTAAATCATCATTTTATCGGTGAAAATGGGCTTGAATATATGATAAGTATGATGGCAGTAGATGCTGGATATGCAACGCAAGAAGTATACAATTGGGTAAGAGGTCATCAGGGCTCTGGAAGAGTAATGGCAGTCAAAGGTGTAAATAAAGCACTAGTACCACTTAGCAGCCCAAGTAGAGTAGATATAACAGTTGGAGGGCAAAAGCTGAAAAGAGGAATAAAGCTCTGGCCAGTAGGAGTATCGATATTAAAGTCAGAGCTTTTTCAATTACTTAATATTTTAAAGAAGAAGAAGGAAAAGCTCTACCTGGATATTGTCATTTTCCCGAGTATGCACCTGAATATTTTAAGCAGCTAACGGCAGAGCAATTAGTCAGCAAGGTAGTAAAAGGATACACCAAACAAGAGTGGCAAAAGGTAAGAGAAAGAAATGAAGTACTAGATTGCCGAATTTATGCGAGAGCAGCATCGTAGCACTGGGAGTAGACAGATGGCCAGAGAGTAAATGGAATAGTTTGAGTGAAAAATGGAAAGTAAAAAATCAAAAAAAATAGTCAAAAGCAGATGGATTGGTGAGCAATAGATGTACGATCAAGAATATTTAGCAAAAGTTGAGCAAGCAATACAAAAGTTACAAAGTGGAGAAAGAGTAGTATCAATTGCATATGGTGACCATGTGGTGAGATATGCAGAAGTAGATATAAATGATTTACTCAGCCTCAGACAACGCATCAAAAGTGAACTAAAGATTGCTGAAATGAAGCCTAAACGAAAGATTATTTTTGCAACAAATAAAGGAGTTATATGAGGGAGAATTTTCTGAGAAAAGTTGTAAAAGAGGAATATTCTGAATTTTTAGAGCAAATAAAAACTTTCATAAAACTATGGGAGAATAGAGAGAGTCCAGTAAGTGGAATAATAGATTTGTCACATTGGCAAGAAAATGTGGATTTTGAGTTAGCTAAAGAGAATGGTATAGTTGGAGTAATTCACAAGGCAACGCAAGGAACAGAGTACATAGATTCAAAATACAAAGAAAGAAGAAAAGAAGCCAAAGACTTGAGTCTATTATGGGGTTCATATCATTTCGGTGTAGGAGAAGATGGAAGAGATCAGGCAAATCACTTTTTGAATACAGTGGGTGAAACTAAAAATACAATTGTTGTGCTTGATATTGAAGAGAACGAAAGTGGAAAAAATATAGAACCAAAACAAGCAGAAGATTTTGTTAAAAAAATTCAAGAAGAAACAGGGCGTTTACCTTTAGTGTATGGAGGTGCTAATTTTTTGAAAGATTTTGCCACACCAATTTTGACGAAATGTCCATTATGGATAGCAAGATATGGAGATCAACCTGTTTTACCGCAAGGATGGAATAAGTGGATTTTATGGCAATACACCGACGGTAAAAATGGTCCAGAGCCACATGAAGTAAGTGGAATCGGAAAATGTGACAGAAATAAATTTAACGGAACTTTGAAAGAATTAAGAGAATTTTGGTTAGTATGATGCTGAAAAATTTCAAACAACTATTTAGTAAGCCAAAAATAAAAAATTCTGCATGGGATGCATCAGGCTCAGGAAGAAGAGTAATGTATTGGCAAGGAGAAACAGGAAGCATAAATAATTTGCTTTCTCAAAGCCTTGAGCACTTGCGTAGCCGCTCTCGTGATATGGTAAGAAAAAATCCTTACGCTGCAAATATAATTGATACGATAGTAAGTAACTCTATTGGAACAGGAATAAAACCACAATCAAAAGCAAGAGATGGAGAATTTCGAAAGAAGGTGCAAGAATTATGGCTAAAATGGACAGATGAAGCAGACAGTAGTGGAGTAAGTGATTTTTATGGATTACAAGCTCTAGTATGCAGAAGTATGATAGAGGGAGGAGAATGTTTTGTAAGATTAAGAACGAGAAAGCTGGAAGATAGATTTTCTGTACCATTGCAACTTCAAGTATTGGAATCAGAGCATTTAGATAATAAAAGCAATCAGACTCTTGCAAACGGCAATATTATTCGAAACGGTATTGAATTTAACCGACTTGGTCAAAGAGAAGCTTATTACCTATTTAAAGAACACCCTGGTGAAAGTATGTTTGGAGAATCAGTGAGAGTACCAGCAAATGATGTTTTACATATTTATAAACCATTAAGACCTGGGCAAATCAGAGGAGAGCCTTGGCTTTCTAGTATACTGCTAAAGCTCTATGAGCTTGATCAATACGATGATGCAGAGCTGGTGAGAAAGAAAACTGCAGCAATGTTTGCAGGGTTTATTACAAGACTTGATCCTGAAGCAAATATTTTAGGAGAAAGTGAAAGTAATGAGCAAGGAGTAGCACTATCTGGCCTAGAGCCAGGAACAATGCAGCTTTTAGACCCAGGAGAAGACATAAAATTTTCAGAGCCATCTGATGTTGGAGGAAGTTATGAAGCATTTATGAGACAGCAACTAAGGGCAATAGCAATAGGTACAGGGATAACATATGAGCAACTAACAGGAGATTTAACCGGTGTTAATTATTCATCAATTAGAGCAGGATTAATAGAGTTTCGTAGGAGATGTACAATGTTACAGCATAACATAATGGTATTTCAGTTTTGCCGTCCTGTATGGGATAGGTGGTTAGCTTACTATTACCCACAAATGTAAATGGACTTATAAATAAAGGATTAGAATATTAAAGAAGGAGCAAAAAAAGAAGTAAAATGGATACCACAGGGATTTGATTGGGTGGATCCGCTAAAAGATCAGCAAGCACAGCAAATGGCAGTAAGAAATGGATTCAAGAGTCGAGCGGAAGTAGTATCAGAAATGGGTTATGATGTAGAAGAAATTGACCAAGAAATAGCAGAAGATCAAAGACGTGCAAGTGAACTGGGCTTAAGTTTTGATTCTGACGTTACGGCCAATCAAGAGGTGATATGAAACAACAATGGCTAAACAGATGTGTAATGGTAGAACCAAGGAGTTTTGAGTTACTGTCACTACAAACAGGAAAGCAGCCTATCTTTAAAAATATAAAACATGCAGTAAGAAATAGTGAAAGAGGAATAATACCGATACATGGCATTTTAACTAAAAAACCTGGTGCATTTGATGAAATGCTCGGAATGACATCATATGAGCAAATAGAAGAACAAATTACACAAGCATTAGCAGATAGTAGCATAGAGACAATTATACTTGATATAGATAGCCCAGGAGGAGAGGTAAACGGTGTATTTGATTTAGCTGATTTTATTTATGAATCAAGAGGAAAAAGAGAATAATAGCGATAGCAAATGATGATGCATATTCTGCTGCGTATGCTATAGCTTCTAGCGCTGAAAAGATTTTTCTCACCCGCACTTCAGGAGTTGGGAGTATAGGAGTAATAGCAAGTCATATAGATCAAAGTGGGTTTGATGAAAAATGTGGAATAAAATATACCACAGTATTTGCAGGAAGTAGAAAAAATGATTTAAATCCACATGAACCAATAACTTCTGAGAGTTTAGAAAATCTAAAAAGCGAAGTGAATCGTTTATATGAAATGCTGGTTGAGCTAATAGCACGGAATAGAAACCTCTCTGTAGAGGCAATAAAAAATACTGAAGCAGGGCTATATTTTGGTGAAAATGCAGTAGAGATAGGCCTTGCAGACGGAATTACAATTCTTTCATATATTAATAAAAACAGGAGTATTACTATGAATGAACAAACTACAACTGACCTAGAAACTGATAATTTAACTAAGTATCGTACTGAAGTTCTTGAATTAATACGTTTATGTAACTTATCACGAATGCCAGAGAAAATAGGAGAATTTATTGAGCAGAGCGTAAGTGTTG

>Phage wAsoc Assembly scaffold 2

AAAAGAAGAAAAGTAGAATTAGCAACGGTAAAGGCATTAAACAAAACGGCACTGTGGTTAAAAGCGCAAGCAGCTAAGGAAATCAGTGAGGAAAAGAAGATAAAATTAACGGTAATGAGAAGAAGGCTAAGAATTTTTAAGGCGAAAACTAGCAGATTAGAAGTGTTAATTAGAGCAAATCTCTATGACATTAGAGCATCGACAATTGGCAAAATACAAAAAACAAGAAGAGGATCGAAAGTAGGAAAGCATGAGTTTATAGGAGGATTTGCAGCAGTTATGCCAAAAGGAAATAGCGGTATTTTTAAGCGTGAAGGAAGAGCAGCATTGCCAATAAAGGAAGTTAAATTGCCATTGGAACCAGAAGCATCAAAAGTAATAAGAGATCTTGTTAATTATGAGGTTGAGAAAGTGTTTAAAAAGTTCTTTGAGCGTGATATTACAGAAAATGTATGAATTTTAAAGATTTACATCAAGCAATTTGCACTAAGTTAAAAGAAGAAATATCAGCAATACAGACATGCGAAATTTATCCATCAATAAGAAAAGAATTATTAGCGCCAGCAGTATTTGTGGAACTTAGTGGCTTTGAAAAAGGATATGATCCAGGAACAGAAGAATTGGCCATGAAAGCAAGATTTGAGGCGCGAATTGTAGTTGATGGAACAGTCGAGAATTCCTCGTTAGTTGTAAGGTCATTAGCAGCAGAAGTAGCAAGAGTAGTAAATAAAAATACTTGGAACGTGAAAAATGTTTCGCCAGGAGAATTTATCTCTGCAGAAGTTGACGGATTTAGACCCGAATTAGATGCATATTTGGTGTGGATGGTTGATTGGAGTCATCAGCTTCATTTAGGTAAATCAGTATGGGAAGAGGGAAAAATTAAGCCACATAAAATAAAAGTAGGAGAAAATGTTGGAAAATAATTTTGCCATTTCAGAGCTAAATAGAAAATTAGCAAACATTATCCGTATTGGTCTAGTAAAAGAAATAGATTATGAAAAAGCAAAAGTAAGAGTGAAAATAGGAGAATTTATAACAGATTATTTGCCATGGATAACAAGCAAAGCAGGAAAAGATAGAGATTGGTCTCCGCCAGATATCGATGAACAGGTTATGGTATTTTCTCCTCTTGGTGAATTATCATTAGGAGTGGTATTAGGAGGAATATATCAGGAAAAATACCCTGCACTAGAGAATAAAAAAGAAATAAATAGTATAAAGTTTCAAGATGGGACAAAGTTTACATATGATAAAGAGAAGCATCATTTAGAGATTGAAGTAGTAGACAAAATAACACTGAAGGCTGGGGAATCGAGCATAGAGATAACAAAGAGAGAAATAAAACTGAAAGCAGATAGAATAAACCTTAATTAAATGAATAAATCGGTTGTACGAGTAGGAGACTACTGTGGAGAAGCTATACCACATTTCTGCATTAGCGGCAGTAATAATGTTTTTGTAAATGGTAAGCCAACGTGCAGACAAGGAGACAGTTTTAGTGAAGGAAAAGTAATGATTCAAGGATCAAAAACAGTGTTTGCAAATGGCTATGGAGTGGGAAGAGTTGGTGACATTGTTTCTTGTTCTAATACAATGAAGAGAGGTAGTAATAATGTTTTCTCAGAATGACTAAAATTGATAGAAATTGGGATAAAGTTATAGAAAATAATTTTTTAGATAAAATTTCTGATTTACCTACACTACATCCTAAAATGGGTATTTTACGTTATCCTGCAAATGTAGGCTGTGTTTTCGGATTGGTAGAATCTGATACATTTAATGTGATATTTGGAGGCAATTTTGATGATTCTAGAATAACTGTAATAGCTAAAAAAATAATATCTCTTTATAGGAAGAAGAATTTACCATATGCATGGTGGGTTGGTCCAAATTCAAGGTCAGAAAGATCTCACGAAATACTAAATAAACTAGGTTTATCACACACAGAAACTGAGATAGGAATGATGATAAGAGTTGACGAAATTAGTGCAGTGAGCTCGTACAATCTTGATATAGTAATGGTAGAAACAGAGCAGGAATTGGTAGATTTTAGTCGTGTATTTGCATCTACGGGTGATAAAGAAGCATTAATATATTATAAGATGGTAGAACCATATATAAAAGATAGTAAGATAAAATTGTTTGTTAAATATTGCAATTATGTACCTGTTGCTACAGCAAGCGTGATATTAAATAAGGGAAACACTGCAGGAATATATGATATTATAACTCACCCAGAGTTTAGATGTAGAGGTTTTGGAACAAGTATAACAAAGTTTGCTTTAAATTATATCAAGGAGCAGGGATATAAATATGCATGTCTTCAAGCCTCGGAGGAAGGTTTATCAATGTATTTAAAATTAGGTTTTGTGCCACATGGTGAATTCTTAGTTTATTCAAATAAAAGAACATAATGAAGGGAATGAGCAAAGAAACAGGAAAAGCGCTTGAAGGAATAGACCATCTGAAACAATCAATAATTGATATATTAACCACTCCTATTAACAGTAGGATAATGAGAAGGGATTATGGGTCGAGGTTATTTGAGTTAGTTGATAAACCGATAAATAGAGATTTAACACTTGAGATATATGCAGCAACAGCAGAAGCACTGGGGAAATTTGAGAGGAGATTTAAGTTAGAAAAAGTAAAAATGACAGAGGTGAAAGAAGGAAAAGTAACGCTTGACCTAGAAGGATTATATGTACCAAGTGGGAAAAACATTCGCTTTGATGGAATGGTTATATAAGTATGCAGCAGCCAAATATTATCGAACCACTGAACTTTGAAGAGATTTTTTCTCGGATGAAGGAAGAGTTAGTGAAGAGAGATGAAAGTTTTACAGCATTAGTAGAAAGTGACCCAGCGATGAAGGTTTTGGAAGTTGCAGCATGGAGGGAATTACTACTCAGAGAAAGAATAAATGAAGCAATAAAAAGTAATTTACTTAAGTTTGCAATGGGAGAAGATCTTGATAATTTGGCTGAGTTTTATGGAGTGGAGAGGCAGAAAGAAGAAGAGGACGAACGATTTAGAAAAAGAATTAAAGCAAAAATAAAGGGGTGGTCAACAGGAGGAAGCAAGGAACATTATAGGTATTATGCACTGTCAGCAGATAGTAGAGTAAAAGATGCGTTAGTTGAGTCACCAGTACCTGGAAGCGTGCAGATCTCAATTTTATCCACAGAGTTATCCACAGTGTCAGAAGAACTACTTGAAATTGTAAAAAAGCAGGTTACTAGAGATGATATAAGGGTTTTAACAGATACAGTAACAGTAATTGGTTGCAATATTACGGAAATAGATATTCACAGCAGAATGAGCATAAGTCCTGTAATATCGAAGGAGGAAATTAAGGAACAGTTCATTAAGAAGTTTGAAGCAAGTAGAAGGTTGGGATGGAATGTTACAAGATCATGGATAATAGCAAATCTATTTGTAGATGGTGTAGAAAATGTAGAATTAATCGAGCCAAAAGAGGACGTTGTAGTACTAGGAAATGAGTGTGCAAATTTGCGAAATTTAAAGATTGAGTAATGTTATTACCACCAAACGCAACAAAGCAAGAAAAAGCGCTGGTTAATGCAATAGATTATAAAGTAGATCCTGGTTGTATAAGGGGGTTTAAATTTAACCTAAAAGAAGAAGTATTGCCGTGGTTGGTTGAAGAGTACGGGCTGGGAGAAATCCTACATTGGGTAAAAGATAAGAAAAGGACTATAAAAGAAGGGGTAAAATTTCAGCGTTTAAGAGGAACCCCAGAGTCACTTAAAATAGCACTAAAATGGGCGAATATAGAAGACATTACAATTATCGAAGAACCACCTGGTAAACATTTTTTTGAACTGCAGATAGGGATAAGGGATGTTCCAAATGACTTTTTTGTAGATGCAGTAGTAGAACTTGCAAAACTATCACTACCAGCAAGATCAAGGCTAATGAGGATTTTCAATGATCACTATAATATTAGCAGATTTATTTTAGATGAAAGCTTTTTTGGAAGTTTGTTATCGGATTACTCAGGTAAAAAAGTTGAAAAAGACGGACCAGTATTATCGTTTGGAAGGGTAAATTTTTTCAGGTTTAGTGGTACATCAATTAAGGTTATAGAGAGTTATCTACGCGACCATTATGAACAAGCATTTAGTAATGATATATATCGGTTAGATGTAGCAGTACTTGGAGAAACAGAGCCTCACACAAAGAATTACAAAGGCATCTATGAAAGAAATCATCAGTGGTACAATTTAAAAGCACTATATCCATTACCACAAAGCTTATTGCCAGAAATTAAGTTTGCTAAGGCACAAATAGTATTATCAGACAGTTGGAATTTAGGAGAAATAAACGCATGTTTTCCAGTAAGTAGTGTAGAGGAAAGAGGGAATAAATTTGTATTAGGAAGCGATAAACTTTCAGGGCAACGTTGGAGTTTAAAACACAAGCCAATTTTAGAAAGGTTTAGCGTTACTCACCATTACAAGGTAGAAGATTTTACCAATCAAAAAGTTATAAGATTTGGTATAGCAGAGCACAATATTCATTTTGAAAGTGAATTAGATTTAGAGCAAAAAGACTCAATACACGAATTAGAAAATTACATTTTAGTATTTTACCCGGGAGTACTGAAATGGCACGAACATCGACATTTGCACAGAAGTTGGAAAAGTGGCCAAGTTATATCTATAATAAGTTAAGTACTTATATTTAGATCCTCATATATTATATTAATAATAGGTAAAAGTGTATGAAATAAAAAGAAAAGGACTTGCTTTCTCATGCTAAAAAGGACATGACTTGAATAGCAGCAGGTAAACATAAGAAATTTATCTGGCGCTAGAAAAGCTAATTTTGTGAGGTATGAAATGAGTTTTAGCAAGGAATCTTTTAGTAAGTTTTTTAAAGAAGTATCAAGTAACGATATTAATAAAAGAAATGAAGAAGGAGAGACGATCTTGCACCAAGCAGTAGAAATCTCCGATTACAAAACAGTGAGGTTATTAATAAAAAAAGGGGCAGAGGTAAATGCAAGAGATAAAAATGGTTATACACCTCTGCACTGTGCAGTATTCGCGAAAAGTTTAGAAAATGTAAAAGTGCTGCTAAGGGAAGGAGCAGAAGTAAATGCCACTCAATATGTCACTGGATGTACGCCACTGCACTCTGCGTGTAAAATGGGAGGAGCAGGAGTTGAAATAATAAAAGAGCTGGTAAAGGCAGGGGCTGAGGTTAATCAACTGAATAAATATGGCGCAACACCAATGTATTACATCTGGGAAAGTGAAAAGTATTGTTCATGGAATAGCGAAGAGAATGAAAAGGCGAGTAAGTTTCTGAGAGAACAAGGAGGAATAACAAAAAGTAGAGAACTGACGTGCTATGGAATAGAGAGGCTAGTGGGAGAAATAGCAGACATGTTGAATGGAAGCTACATGCCGGAGCTAAAAATAATAGAGATAGGAGAAATAAGGAAGAGAGACAAATCGCTAATAAAGAAAGAATGTCAAAATTTAGCAAGCAAGATAATGAGCCAAGTAAACGAAATGATAGATGAGGTGGTGAGAAAGAAGGCTTAAAATTTAAAGAAAAGGTGAGGTAGATTATGTTAAAGCTTGGCAAATTTAATAAGTCAGCAAAAGAATTATTAGAGAACTCATATAAAAATATTTATGCAAGAGACGAAAAGGGAAGAACAGTTTTGCATTATGCAGTAGATGCAAAAACAGTGGAGTTATTAGTTGAAAAAGGAGCGAATGTGAATGCAGCAGATGTAGAAGGATATACAGCACTGCACCTAGCGGTAACGGAGAAACGTCTAGAAACAGTTAGAGAATTGATAAAATCAGGAGGGAATGTAAATGCTGAAGAGTATGGCAATAAATGTACTCCATTGCACCTTGCATGTATGGTGGGGAAAGTGGAAGTAGTAAAAGAGTTAGTGGAAGCAGGAGCAGAAATAGAGCAAGAAGATAAGTTCGGAATGACAGCAATGGATTATGCGAAAAATAGTAAAGAGATAATCGAGGTATTAAAGAAAGAAACAGACAGAATTGAGAAGTTATTTATAAGAGGCTGAAAACATGGAGGAAGAAACAGAAAAGAAAGTAATGAATTTAGAGAAAAAAGCGTTAGTGGAGTTGAGAAAAATGTGGAAGAAGGTATTTGGGGAAGAGGCACCTAAACATTCAAAGAAATATCTGATACCGAAACTAGCATACAGAATACAGGAGGAAGCGTATGGAGAAATGTCAAGAAAAGGAGCAAAAAGACTAGAGTATTTGGCAGATCGGCTAGAGAAGGGAAAAAGAATAAGTAGTGATAAACTGCCAGTAGCAGGAACAGAGCTAATATTAGAGAGAGGTGAAGAGACTCACGCGGTAATGGTAACAGATAAAGGTTTAATCTACAAAGAAGAATTTTTTACGTCATTGTCAGCAGTAGCCGGAAAAATAATGGGAATGAGTTACAATGGCCCACTTCTATTTGGAATGAGAGAAAGGGAGAAAGAATGCTAAAAGAGGTAAGATGCGGAATATATACGAGAAAATCAAATGAGGATGGGCTAGAGCAGAAGTTTAACAGTTTAGATGCGCAGCGAGTAGCATGTGAGAAATATATAAAGAGCAAAGAAGGCTGGGTAGCATTGGCAAAAAGGTACGATGATGGTGGATATTCAGGGAAAAATTTAGAAAGACCAGCGATAAAGGAATTATTTGAAGATGTAAAAGGAGGAGAGGTAGATTGTGTAGTAGTATATACGCTAGATAGGCTATCAAGAGAAACAAAAGATAGCATAGAAGTAACATCATTTTTTAGAAGGCATCGAGTAAATTTTATAGCAGTAACGCAGATATTTGATAATAATACGCCAATGGGGAAGTTTGTACAAACAGTATTGTCAGGAGCAGCACAACTAGAAAGAGAAATGATAGTAGAGAGAGTAAAAAACAAAATAGCAACATCAAAAGAGCAAGGGCTATGGATGGGAGGAACTTTACCGCTTGGATATGATGTAAAAGATAAAGAATTAATAATAAATGAAAAAGAAGCAAAGACAGTAAAACATATATTTGAAAGGTATGTGGAGCTGAAGTCAATGGCAGAACTGGCAAGGGAGTTAAATAGTCAAGGTTACAGAACGAAAGCAGATATCTTTAAAAAGGCAACGGTGAGAAGAATAATAACAAATCCAATATATATGGGAAAAATCAGACATTATGAGAAAGAGTATGAAGGAAAACATGAAGCAATAATAGGAGAGGAAAAGTGGAAAAAAGCACAAGAATTGATAAAGAACCAACCATATAGAAAAGCAAAATATGAGGAAGCGCTACTTAGGGGAATAATTAAGTGCAAGAGCTGTGATGTAAATATGACGCTAACATACTCAAAAAAAGAGAATAAAAGGTATCGATATTACATATGTAACAATCACTTAAGGGGAAAAGGTTGTGAATCAATAAACAGAACTATAGTAGCGGGAGAAGTGGAAAAAGAAGTAATGAAAAGAGCCGAATACTTATATGAAAAATGGGGAGAAAAAGCGGAAGAATGGAAAAATTTAAGTTTTGGAAAACAGAAAGAAGTAGTGAAAAAGTTAATAAAGGGAGTAATGATAAGAGAAGATGGAATAGAGCTGAGTTCAGAGGATAAGGTAGAATTTATACCAATAAAAAAGAAAGGAAATAAATGCATAGTAGTAGAACCAGAAGGTAAAACAAATAATGCGTTACTCAAAGCAGTGGTAAGAGCTCATTTGTGGAAACGGCAACTAGAAGAGGGAAAATATGGAAGTTTGAAGGAGCTGAGTGCCAAAATTAATATAGGTACAAGACGTATACAGCAAATTTTAAGGTTGAATTATTTAGCTCCGAAGATTAAAGAAGATATAGTAAATGGGAGGCAGCCAAGAGGTTTGAAGTTAGTTGATTTGAGAGAAATACCGATGCTGTGGAGTGAGCAGATGGAGAAGTTTTATAATTTGATTATTATGTAAAAGGTATTGACTGATAACCTTATGATAGTTAAAGTATGTTATATTATATAGCTAGAGAGGTAATATGCCGAGTTTTTTTGATAAACCGTTTACAGAACAAGTAGAAGAAATCATTAAGACGGGAGATCTAAAACTACTGCAGGCACTAATTGGTACAAGGGCACCAATTGGGTTATCATTATATTGTTCTAGCTTTTTTCTTACTAGAACAGTTATTGCACAAGCAAAGTATCAAGCATATAGTGCTCTGCTTAACGTGCTAACTGAATATCAAAAAGCAGAAAAAGAGATTTTTGTAGACAACATAAAAAGGGATATAGAAAGACAAATAAGCGGTAATGAGGATAGATTACGCCAAAAAGCACTTTTACCATCGTCAAGTAATAATTTAAAATCTCTAACTAATAATAGTTGCTCTGATTTAGAAAGTTATGTTCAAGAAACAGATGTATCAAGTTTGTGTACTTTTAACTTACAAAAAGTAATTGAAATTTATCATAACTATATTTCTCGTGAAGAGGTAAAAAACTTAGAGAAAGTACTATTAAAAGCCAAGGAAATGGAAGATAGGTACTCGTATATAGAAAGTCAAAAAACAAACTTACAGTATAATAGCAAAAGAGAAAAAGCACGTAATACAGTTAAGGATAATGAACCTCTATTGCTCAAAATAAAGGACAAGACCTTTTTTGATAGCTGGTATACAAAAAGTTATTGTACGAAGTTCTTTGTGAAGCAGAAATGAATAAGAAACATATACTACATGAAGAAATAGTAAAGAGCAGTTCTCCCTATATGAAAGATAAAATTAATAGTGCAAAAAGCAAGCTAGGTACTTCCTTTAATAAAGAAGCAGTTGGAAGAGTAATAAATAAATTAGTGAATGATTACCTTAAGATTTTACCAACTAGTTTTGGAGTAAAAGAATCGTTATTGTCCTACATGAAATGTGTTGATATTGATGATTATATAGGAAGAATGAATTATGAGGCAGTAAATCATAGGATTATTAGTAGAGCCAATGAGGTGATCTTAAGTGAGTCAATTGTACAAAATAATTTGAATGATGATGTAGAAAGAGGTTTACAGTACATAGTGGACAGAATGGATGTATCTCTTCTTACTGCAGTAGAGCAAGAAATGTTGAATGAAATAGCAGAAAGAGCAGCAAATCATAGAGAAAGACACAGTAAGAGGAAAGATTTAGAGTTGCTATCTATATGCAATGGAGGAGAATTTGAAGATAAGCTTTCTTTACCAACGGACTTGAGTTTTTCAGAGATTGAGCGTTTATATAAAGATATGCTCTCAATAGAGTTTAAAGAGCAAGG

>Phage wAsoc Assembly scaffold 3

GCTAATTCATCTCTTCTCATAGATAGATCCTTAATTTCCTCAAAATGTGCCCAAAGATGATGTCCTTTGACTCTATCTATGCCGTTTTTTATGGCCATCTTAACTATGTCATAGAGTTCATGTAAATTACTTTCCAAAAATGTTAATTGTAAAGTAACGGTACACCTTTCACCTGTATCGCTAAAGTATTTATCTCTAACTTCAAGGAAAGTTTTTAAATTTTCTGTCACTACTTCCCATTTTGAACCTTTCATGATTCTTTCATGAGTTTCTTTAGTTGCCCCATTCCAGGAAATCTTAACATCAGATAAGATTGGTACCAATAGTTCAGCCCATTTTCTAGCTCCTTTAATAGGAAAAGAACCATTGGTTGTTAGGTTAAGCTTCAAACCAAACTCATGGCACAAATTGATTATTTCATCAAAGCTTTTGTACATTAAAGGCTCACCCATAGTAGAAGGAATGATTTCCTTTAAAGGTGTGCCAGCTGCTTCCTTAATTACTTTTCTGATAGTTTCTATCGACAATATCTTAGGTTTTATTCCTTTAGCTTTCTTTTCTTCTTTAACTTTGCTGTATGGTGAAAAACATTCACACATTATACAAGCAAAATTGCAGTAATCTGGATTAGTATCAAAAGTAATTCTCCAAGGACCGGGTTTCACAGCTACTGATTTGCCTTTCTTTTCAATAGCATTACGGTAAATCTTGTTAAGTTTCTCAGTATGCTCACTTATAGAAGGTATATTTCCATTTTCAGTATAAGGATAGCCTTTTTGGGTAAGTTTATTGTATAACTCCTGATTTGTAGATAGGATTTGCATTTTTTCTGATAAACTACTTGCATCTCTGTGCTTAAACAATAGTCCATCTCTTACATATTCTGCCATGCCACCATAATCGGCTGTTATAACTGGTATTCTAAGTTGCTGTGCTTCATGTATTACTAACGGTGAATTTTCACCCCAAATTGAAGGAACGACTATTGCGTCAACTTTATTAAATACGTCAGTAACTATATTCTTATTATCATAACTTCCCATCCACTCTATTCTTTCTTTAACAACAGGTGAAAACTGATCGGCAATCGCTTTTAAAGCTTTAGTTTCTTCTCTTGCTGCTCCCCAAATTCTAAGTTTTGCTTGAGATGATAAGTGAGAAAAAGCTTTCAATAATAGATCAACACCTTTTTCGGGAGTATGAGTACCAATATAACCGAAAATAAACTCTTTTTCTTGTGCTCTATTTCTATTCTTAAGACGATTGAGATCAAAACCATAATCAAGATAAGAAATTTTGTTTATCGGAACATAAAAATCCTGAGTAAATTTATCCATCAAGAATTTCGATGGGGAAATAAAGTAATCTATATAGTCTATTATTTTCCTTGTGTGCTTCATTCTAGTTGCAACCCATTGTTCCCAATAATTTATGTCTAAATTCAGGAATTCTTCATCTCCTGTAAAATATCCTTTGTAACATTGAGTTGCACATTTTTGATCCTTTTGTCCATCACAAAGCCGTAACAAATCTTCAGAATTGCGTTGAATAAACCTTCCTCTTGGACACATTAACCAAAAGTCATGTAGCGTAAAAATTGTAGGTATGTTTTCTTTAAAAGCAATTTTTGGTAAAGTAATTGATAGATGATTAAGATGACCAAAATGTATAAGATCTGGCTGAAAGTTATCTATTACTCTTTTAAATTGTATATCTACTTCTTCATTGATAAATTTGTAACGATATTTAGCTGTGGGTATATTAATTAAATGCAGTAAGATTCGAGAGTCACTACTATCTAGGACTGTAGTGTAATAAAAATCAGGTAAAAAGCTATTTTCATACCTAGTAAATACTTGTACCTCATTGTTATTTGCTAATTCATGAGCAAGAGTTTGGCTATAAACCTCTGAACCAGCACTATAATAAGGAGGGTAACCATGAATAACTTTTAATATTTTCATATATTACCATATCTATATTGTCAGGATATTCTTGTGTATTGTTTTGTAGACTTTTTATAAAATTACTTAATCCTACTTCTAATGAATGTTTTGGAGACCAACCAAGTAGCTCTTTAGCTCTAGTAAAATCACCATGGAACTTAGTTACATCAAAATTTCTTGGAGGATAAAAATCAATTCTAGAATCACTTTTCGTGACTTTTAATATTGTTTTTGCTAAGTTTTCTAAAGTACATGGGCTATTAGTAGTAAGATGAATAGCAGGAAGAGAAGATTTTTCGCTTTGTAAATATTTAACAGTTAAGCATATACCCTCTATAACATCATCCAAATAGGTAAAATCAAAAACACATTCTTTACCTTCTATTTTAATTGGATCACCTCTTAATGCATTAATACAGAGTGCAGGAATTACTCTACTGTTATGATCTAGTAAACCACCGTATACATTTGAAAAGCGTAATATTGCTACATTAAAATCTTTTGAACTTGTTATTTGTTCCTCAATAAATGCTTTACCTTTGGCATAATTATTTATTGGATCGATACTAGCAGATTCTGTAACCGGCAGTTTCTTCTGCTCTCCATAGACTTCCCTACTACTTGCATATATAAACCACGGTTTATTTGAAAGTGACTTACATAACCCTAAAAACTGCATTGTACCATCAACATTAATTTTTTTACACAGTTCAGGATAAAGTTCACCATGTATAACTCGAGAAATTGCAGCCAGGTGAATAATTCCTGTACACTTAGCAAGTAGTGGTACTACATCTTCTGAAAAAAAACTGAGTGGATTATCGCGAAACCTAATATCGCAGCTTATTACTTCATGGCCCTGGTTTTCTAACTTTTTCACCAAGGTTGACCCTATCAAACCTGCAGCACCTGTTATTAATATCCCCATGTATACCCTCTCACTACAACTAGGAAAATTTGTCGTACCAATTATCTTACAAGTAAGAATCAAAAGGTATTATAAAACCAGTATAAAACAAATGTCAACTTAAATTAATGAATAAAAAAATGTAGTTATGAAAGAAAAGTTGGCCGCCTTTTTTTTTGAAAATAGTGTTCGGCTTTAATCAAATCCTCTTTATTATTTACTCCCATTGCTTCTCTTTCATCAGAAACTACATAACCTACACTCAAGTTATTGTTTGCTGCAATGGGTACTATATCATTAAGACAATATTCATTGGTCGAATTATTAAATTTTATTTCTTTTACTAAGGTAAAAAGATCTTTAGCATATGAAACTATTATTCCAGAGTTTGCAAGAAGCATTTTATCTCCATTTTTTAGGATTTTTTGAACATTTCCGTAATTATCAATTACTAATCTTCCATATTGTTCATCTTGACTATTGAATCCAAGAAGAACTAAATTATTATACTTTAAACAATCAGTCATTTTCATAACTGTATCACTAGATATAAAGGGAGTGTCTCCATACTGTATTAAAACTATGTCTTGATCAGATAACTCTTCCAGATTTTCTAGAGCAATTTTAACTGCAGTGCCAGTGCCTGTAATATCTTCTTGCATTATTAGCTTTATATTGTATTGATCGATTATACTTTTTAGGGTATCAAAGTTTTCTAAATCTTTTAGAAGGGAGTTGTTAACAACAATTGATAAGCTTTTTAGACTCAGCGATTTTGCATTAGAAATTATATGTTCTAAAAGAGTAAGATTTCCCACTTGGTGCAACGCTTTTGAATATTCAGAATTCATTCTACTGCTTTTTCCTGCTGCTAATATTATCAAAATTTTACTCATACATAACTCAGGAATTGATTAGGTAACAAGTTAGTTTATAATTAGTAAGATGTAAAGCCCACTGTAATTTTTTAAATGATCCAGATCTTGGCACCCTCATTAGAGTTTCATTTAAGAGAGCTATAACTTTAAACTCTTTACTCGATTTTCTACGCTGACATCTTGTAAACTCTGATCTGGACTTTTTAATATTTCTAATAATCTACTTGCAAGGTTTGAGTGTTTATTGATTACTGCAACCTGGACTAATGACTTGCCTTGATCATCACGAGCATTTGTTACAGCTACGCGCTCATCGGGTTTTATTGCTCTCAGCTTACTGATAATTTCAACATTACCATTTTTTGCATTTTCAAATAGCTCTTCTACTAATTTCAATAGGTTAGTAATATTTTGGTCTCGAGAAAGATCAAGTGGTGCTTTACCTTCTTTATTCTTGATGTTATAAATTGCACCATGTTTTAATAAAGATTTTACAACTTCCAAGGAGCCACCTTTAGCCGCAACATGAAGTGATGTAGTACCACCAGAAGTTGTTCTAGCATTAATAAAGTCATTTAATTTATCACGACTTACATATTGTAATAAAACTTCAACAACTTCTCTGTAACCTTTGGAAGAAGCTATATGTAATGGCGTATTGCCCTTGTTAGTAATTTGAGCAACTTCAGCTCCATTTTTTAATAAGGTACTTACAACATCTATGTTGCCGTTGTTAACAGCATGGTGTAATGGTGTTCTTCCATCGGTATCTTTGAAGTTAACAATTACCCCTCTTTTGATGCAATTCTCAACCGCTAAAGGATTGTCATTTTCTATGGCCTCAAGTAACTTTTCAGAAAAAGCAGACTGTAACCTCTGATTGATTGACTCTAAAAACCTTTTTACAGGAATGTCAATATTAAGTTCAACCATTTCTATGAACGATCTTACATCTTTCTTTAACTCACTATGCAATAACTTATAATATTTTGTACCTTGCCCTTTGGAAACGAAGCATTCAGCCTTCCCACCGTAAGCTTCATCTGTATACCCTGACGTTATGTGTTTTGGATCAAATTCTAGTGCTACTAGCATATCATTTACTGACTGTATGCTTTTATTATTTATCGAACCAGGTACACAATCTTTGTATACCTCAACTAAGCGATATGCATTTCCTAGGTAATAAGAACCTTCCTTAAGAGTTTTCCAAGGAGAAGTATTCTTTTCTGAACTATATGGTTGCGACATGCTACGCAACATAGGAAGGTAAAGCTCAGAAAAACGGATATGCACCTCTGTAGCACTTTTAAGCTTCTCAAGTTGATCCAACTCACATTCAGGGCTAAACTGTGATTGAACTTGATCTGGCTCCTTCAGCATTTTTAACAATTTAGTTGCAATATTCTTATGTTTATTGGCTATAGCAACTTGCAATAATGTATTCCCTTGATTATTACGAGCATTTGTTATAGCTAAGAACTCATCAGGTTTTACTGCTCTTAGCTTGCTGATAGATTCAACATTACCATTTTTTATATCTCTAAACAATTCTTCGATCAATTTCAGTAAGTTAGTAACTCTTTGGTCCTTAGAAAGATCAATAGGTATTTTACCTTCTTTGTTCTCAATTTTATAAATTGCACCATGTTTTAATAAAGATTTTACAACTTCCAAGGAGCCACCTTTAGCTGCAACATGAAGTGATGTAGTACCACTAGAAGTAGTTTTAGCATTAACAAAGTCATTTAATTTATCACGACTGATATGTTGTAACAGAACCTCAACAATTTCTTTGTAACATTTGGAAGTAGCAGTGTGTAATGGTGTATTACCTTTATTAGTAACTTGACTAACGTTAGCTCCATTTGTTAGTAAGATGTTTACAATATCTATGTGTCCATTGCTAACAGCGTAATGCAACGGTGTTCTTCCATCAATATCTTTATCGTTGGCATCAGCTCCATCTTTTAACAGACGCTGAACAGTTCGTATATCACCTTTACTAGCAGCAATGTTGATATCTTTCTGCAGATGCTGGAGAACCTCTGATGCTTCGCTACCCTCAAGCTTAAATCTAAAATTAATCATCTCTATCTTTTTTAAAATATCTAAAACACTCGGATGACTTGGTCCAAAAACAGCTTTTCTTTGATCTAAACTTTCTCTGTAGACTTTAAGTGCGCTGATCCATTTCCCTTGAGCAAAAAGTACATTTGCTATGTTGTATTGAGTATTCAAGGTTTCTGGGTGATTTTGTTGTAAAGCTTTTTTCTGGATATTTAAAACCTCTTGAAAAGCTTTCAAGGCTTCATGATATTTATTTTGATTGAAGAGTACCCCAGCAATGTTATGTAACGTTCTCAAGGTATCAGCATGATTAATACCTAAAATTATTTTCTTCTTTTCAAAAACCTCTTTATAGATTTTTAAGGACTCTTCGTATTTACCTTGGTTAGCTAGTACCATTGCTATATTATTTTTGGCACTCACAGTGGCTAGATCATGTGTACCTAGTGTTTCTTTCCTCTTTTCAAAAACTGCCCTATTGATATTTAACGCTTCTTCATATTTTCCCTGTTTATCTAATACTAATGCCATGTGAAACTGAGTATTTAAGGTATCTGAATGATTTGACCCTAGTATTTCCTTTTGTCTCTGATAGACTTCTTGATAAATATTAAAAGCTTCTTCATCTTTCCCCTGTCTATGCAGCACTAAAGCGATTGTACTTCTTGTACTTAAAGTATCCTTATCGTTTAAACCCAGCATTTCTTTTTGTTTCTGAAAAATCTCTTTAAGCATATTTAAAGCTTCTTGGTAAATTCCTTGCTTATATAGCACTTTGGCTATATATGTCTGAATATCTAAAGTACCAGGATTGTCTGGTCCTAGTATTTCTTTTCTTCTTTCAAATGCGCTTCTGAAAATACTTAAGGCTTTTTGGTAATTGCCTTGATTCAAGAGCACTAAAGCTGTATCAATCTGAGCGGATACGTCACTTTGCGATATCTGCTTCAACTGCTCGACTTTTGAAAAGTTACTATGTACTGCAGCAACTACTAACGTTTTGTTCTCTCTATTACGAGCACTCATCACTGCTTTTACTGTATCAATATCCTTTATCTTATTTAGGTCATTAATAACTTGAGCATTACCATCTTTAACCTTTTTAAATGATTCACTGACTAGTTTAAATAAGCTAGTTATACTTTTATCCACAGTAAAATCTGATGGTGTTTTCCCACTGTCAGAGACAGCATTGTATACTGCACCATTAGACAATAGGGCCTTTACAACTTTTAAGTGAGAGAACTTAGCAGCATAATGCAGAGGAGTAAACCCCTTGTTACCAACCACATTAGGATTAGCTTTATTTTGTAATAGAATATTGACAACTCCATCATAGCCCTTCCATGCAGCATAATATAATGGTGTTACACTATCAGCATTTTTGGCATTAACAAACGCTCCTGCTTTAATGTAATTCTCAACCTCTGAAGAGCTATTACGTTTTACAGCCTCAAATAATTTCTCAGTTGATGCTAATAGATTGATAACGTCTTTATCGTTAGTCATTTCTAATGGTCTTCTACAAAGCTTGTCAACAGCGTTATAAACTGCACCATTTTTTAATAAAACTTCAATAACGTCTTTATAACCAAAATTAGCAGCAATATGCATAGGAGTTAAGCCATTAGTATCTTTAGCATTAACGTCAGCACCTTGTGCTATCAAATACTTTACAACTTCTAACCGACCTTTCATTGCAGCATAGTGTAGTAATGTCTGGTTAGCTGTACCAAGCTCATTAATACTCAACCCCTTGCTAAGGAAAAACTCTACGGTATCTTTATAACCTTCTCTAGCTGCAATGTGTATAGGCTTTGATCCAGAGGCATTTTTAGCATTAATATTAGACCCTTCATCTACTAGACATTTTACCATTTCCAAGTTACTTTCCTGTGAAGCAATGTGCAGTATAGTCCAATCATCATTGCCTTTAGCATTCATATCTACTTTTTTATACTGCAGTAACATTTTAACCACTTGCAAATGACCATGTGCAACTGCTAATTCTAAAGGTGTTCTGCTCTTATTATCCTTAACATTAACTTTTGCTCGATTACTTAATAGGACTTCAACAACATTAACATGGCCGTGTCCTGCAGCTATATGTAGTGGAGTACCTTCAACATTTACAAAGTTAACATTAGCCTTGTTTGCTATCAAAACTTCAGCAACTTCTTCATGGCCTTCTTGTGCCGCAAGATATAATGGTGTTATACTATTAATAGTCATAGCATCAACATTGGCTCCCCTTTCTATCAGAGCATTTACTATCCCCTTATGTCCTCTTTTAGCAGCCAAATGTAATAGTGTAGCATCTTCAGGACCTTTTATATCAATATGGGCCTTGTTTTTTAGCAAAATCTCTACAATATCCCTATAACCAGCGAGTACTGCTAATGATAAAGGTTCACCACCTTCTGCATTAACACTAGCTCCATTTTCTACAAGAACTTCAACGATCTCCTTATGATTATGCTTAATAGCAGAAAGCAATGGTGTTAGGTTGTTACTCTTAACATTAACACTGGCTCTATTTGCTACCAAAATTCCAACGATTTCCTTATGACCTCCTTCCACAGCTACATGTAATGGTGTACTACCTGCAATACCTTCTGTGTTAACTTTAGCTTTATTTTTTATTAAAAGATTCACAATATCTTTGTGACCTCCCACAATAGCTGCATGTAAAGGTGTAAGACCATAATTAGCACTAGTATTGACTTCAGCTTTACTTTTGATTAGAAAAGCAATAGCATCTTTATGTCCACTTAGAGCAGCTACATGCAATGGAGTTATGCCATCATTAGTTCTAGCATCAACTTCAGCTTTATTTTTTATTAGAAGATCAATAATATCTTTGCTACCATTCATCGCAGCTGCATGTAATGGTGTACTACCCTTAATGTCTTGAGCTCTAACTTCAGCTTTATTTCTTATTAGGAGTTCAATAATATCTTTGTGACCTTTCAGAGCAGCTACATGTAATGGTGTTAGATTATTATTGGCTTTATCATTGATTTCTACTCCATTTTTTATTAAAAGCTCAGCAACTGCCTTATGACCACTCTCTGCTGCATAGTGTAATGGCGTAGCATTATTTTTATCTTTAGCGCGAATGTTAACCCCATGCTCGAGAAGAGCAACAACTATCTTCAAGTGGCCACTCTGCACTGCAAAATGTAGTGGGGTTATACCTTCCACAGTGGCAATACTAGCATTTGCCTTATTTGTCAGTAAAGCCTTAACAATTTTCTCATGTCCATCTTTTGCTGCGTAGTGCAAAGGTGTGTTATTATAGGTTTTGTCAACAACATTAACATTAGCTCCATGCTTCAATAAAATATTAGCTATCTTTTCATGACCGTTTTCTATTGCATAGTGTAATGGTGTACAACCATCTATAACTCTGGAGTTAACATCGGCTCCTTTGAGAATTAAAGCATTCACTACTTCTAGGTGGCCATTTAGTGCTGCTGTATGTAATGGTATTCCCTCCTTATCGTTTCTTGCATTAACATTTGCCTCATTTTTCAGTAAAAAATTAACTAGCCCTAGATAACCACTTTCTGCAGCTATATGTAGTGAAGTAAAGCCACCCATAGTTTCGTTGATGTCAACATTCGCTTCTTTTTCCAGCATAATCTTAGCAACATCAATATGATTGTTTTTTATTGCATAGTGTAAAGGTGAAAAACCAGCTATATCTTTGGTATTAGTATTAGCATTATTTTTTAGTAGAATTTCAACAGCATCTTTGTGACCATTTTTAGCTGCAATATGTAGTGATGTTTTGCCACTATTATCTAGATCATCAACATATACACCTGTTTTTCCTATAAAAAATTCCACAATATTTTTCCTTCCATATGCAGCAGCAATATGTAGTGGGCTTTGACCATTAATATCTTTAACATTAACGTCTAAATTTTGATTAAGAACAAATTTTATAATTTCAAGACTAGGTCCCTTAGCAGCAAAATGTAATGTAGTCCATGAGTTAATACTTCTAGCATTGATGTCTGCTCCTTTTTTGAGATAACTCTTTAAGTCTTCAAGATTTCCTTCTTCCAACGCAGCAAACATTTTTTCCTGATTAGTAATAGTGATAAGGTTTTGATCGTATTTATCTTTCAGCTTGGAAGGATCATCTCTTACCGACTTGCCAATTTTCTTCTTACTTTTTATTACATTTTCTGAAATAAGTTTTTTAGCGTTCAAGATAACTGCTATTGAGGGATCAGATAACAATACATCAACTAAGGTATTATCGTGTGCTAAATGATTACGCAAACATTTTCCAGTTAATAAAGGAGTGTTATCATCTAAAAAAAGTAGATTGTTTTCTAGATGCTTCTCTGATCTACCTAAAATTGACATTATATCCAGAACAAGCATTTCTACTACTGCTTGCAACTTTTTATTCCTCTTGTAGGAAGGAAGCTTTTCAGTTAACTTGCCACTTAATGCGTTATTCCTCAAAATACTTTTCAGTTCAGATAACTTCAGTTCAAGCTGGTTATTGTATTTTTCTTCTGTTATGTTGTAAGCCTTCCTTTGTTTATGTATAGGAATAAACGAACCTTTCTCATTCAACTTGTTTCTTAATTCCTCAATCCATTTAAGGTCACCTGTTCCAAGTTCAGCAATGTAAAAAATCTCACAAGTTAATCTATTTACTTTATCAAGGTTGTCATCCTGTATTCTTGACCTAACACTATGAGAAATTTTCATTGATAGTTCGGCTATTTCTTTAAGATTGTGAGACTCTATTTGGGAAGGAATATTCTCTAGTGTTTTATTAGCAAAGAATTTTATTCCTCTAATAACATTATGGTCAATCTTATTGTCATTCAAACCAACATCTAAGCTCTTTAATGATATAAACGCCGTAACATAATCAGTTCTGATATTTTTTGATTTAGTTTTTGCAAAATTGATTATGTTATCAATTTTATTAAACAGCTCCTTTTCATAATTCGTCTTTTCAGTTATATTATTGCTTAATTCTTTTATGAGTTTTTCAAGCTTATCATGTTCCATCACTTTAAAACTCTCTGTAATCATTTCATCTAATTCAACGTTACTAAATATTCCAGCAACTTCTTTTATCTCGTCTAAGTTTTCACTTCTGGTAATTTTCTTTAGCAATATTATGACTATTTTAATCTTGTTATTGTAAAGAATATCAGTAATTACATTATCGATTCTTTTAGTATCATTTTGAACACCAATAAAAAAGTTAACATCTGTATTTTCCTCAATCTCTGTTCTTTTAGAGAGCGAGTAAGCATGTGATAGTGAGTTACGTAAATCTATAATGACTTCTCTTGTGTTCTTTGGTAATGATAGTAGAAGAAGCTCGCTTGTAGTGCTAGATAACTTGGGAGATTCTAGAGTGTTTTTTAAATATTCACCAATAACCTGTAAAACCCTTGTAATAATTAACTGTCCTTCCCTTTCTTTAGGATTAGCTGATAATGCCAACTTTATATAATCACTTATTTTTTCTAAAGAATGAATATCCCTAATTTGTTGATAATTGCTATACAACTCTCCAAATTGAGGATAATTGCTAACGATCTCTGCAACAACCCTCTCACGTTTTAACTTCGGGAGATCAGCAAATTTACCAATGTCCACACCTTCTATAATATCTTTTTCTTCTTTAAGTTTCTTTGCAAAATTTTCCAAGTGGTCTAGTATTTTACTTTTATTCAATGTGGCATTATAGAATAGATTCATTTCCTGTCGTTTTATGTGAGAAGAAACAAAACTGATCAAACAAAATTCCATTTCTTCCCAAGGTAGTCTATCGTAGGTTGATTTCAACTGCCGCTTTAATATATGAATATTTTGTGCAATAAATTTTGCTATGAATAAAAACTTTTCATCTACTTTTTCTGTGTTTGAATACTCTGCCTTTAACAGGCTAATATTTTGAAGTACTAACTCAATCCGCTCTCTTATGTTATTAAGATAACTCTTCGCATTCTGATCTTGTCCAGAAGCACTAGAGAAAAAACGGAGGTTTATCAATTTATTTTCAACAAAAATTTCTATCTCTTCTGATAATAATACGTTCTTTAGCTTTAATTCCTCATAAGCTCGTGAAAGAATTTCATCTAATTCCCTGAAATGAACAGCAAAATAATTACCTGGCCATT

>Phage wAsoc Assembly scaffold 4

TACCCTTTCTTCATAAGCTACAAGATCTTTCTCATTCCAATGAAATTTATCTAACTCATCATATGCTAACTTTATTATTGGTGATTTTTCTGCTATTTTTCTTAAATCTTCATCAGTTGTTTCTTCTGCATACTTAAAGAAAAAGCACCATCTCTCAACTGTAGTTTCCAACTGCTCTACTTTACTTTTTGTAAATTTAGGTAGTTCAATGAAGACAAATTGAAAATCTTTTAAATAATGTCCGTTAGTCTTTATATCGCGTATATTATGAGTAGAAATATAATAAACATCTGAAGGAAAAGAGTACTATTGGAAATAGCAATAAAGAAAACAGTTTTAAATCAATGTAATTGCCAGACTGTCTTGAGTAAGCTTTAGCAGCATAAAGTTGAGCACGTTTTTCAAAGCCTTTATCACGAGCGAGCTGCATTTCCGCAATATATCTATTTCCGAGAGAATCCTTACAGAGGACATCAACGATACTTTGTTTATCAGAAGCAATCTCAGGATTCATAATAGTGCTAAGGAACTCAACATCTTGAATAGCATTGACTCAGTAAAGCCTAAGATATCATTTAAAAAATGGATAAGGATATTCTTATTTTTTTCAGTACCAAAAATTTTCTTAAAAGTTAAGTCTAATTTTGGATCGAGAAACTTCGAAAGAGCCATGAGAAAGTAAGATAAAAAAGCATTAATAATTATACACAATTGTGAGGAAATGTTCAATCTTTTTATTGTTTTAAATGTGTTGATGAAAAAACGTCTGTCAGAGATCATCTGAGAATTGCATTTAAGGTTTTAGATGGGATTTGGATCATCTTTTTATAATTGCCTTATAAAATACCTTAAAAACCCCAGCAAATGAATTGATAAGCTATGAAGAGCTTAAAAATATTTATTTTCAGTGTGAGAGATTAACCTCCTAATAAAAATACGAGCCCAAAAGCTCAATTACGATTTACCTAACATCGAGAGGACAGGTTTGTCAATCTCTCACACCTTACATTTTAAAATATTCAAAAAATAATATCAACATATTTCGGTAGCAAATTTACGTTGAATCTATATCCTGTAAGTATGGGCGAAAAAAAGAAGGTTACAATATATACAGATGGAGCTTGTTCTGGAAACCCTGGTCCCGGTGGGTGGGCAGCCGTAGTCATGTATGAAAATAAAAGTGTCTTTATCAAGAAACGTATCTCTGGAGGTGAAGAAGACACAACGAATAACAAAATGGAGTTAAAGGCTGTGATTAACGGGCTAAAGATGTTGAAAATTTCTTGCGAAGTTGTTTTACACACTGATAGTCAGTATATTAAACAAGGTATAACAGAGTGGATCAATAAATGGAAGGTAAATGGTTGGAAGACAGCCGATAAAAAGCCAGTGAAGAACAGAGAATTATGGCAGGAACTAGATGAAGTTGCTTTGCAGCATGATATTAATTGGAAGTGGGTTAGAGCTCACAACGGTAATATGTACAATGAGGAGGCAGATAGACTTGCTAGAAAGGAATCTAAAAAGCTAAAATATAGAGATTGTGAAGTCAAGAAATCACCAAAAAATAGAGGGAGCTCTAAGTTTCATAGATTGGGTGGGGAAATATGGCAATAATTCTTTTAGACACAAAAACTATAAATCGTATAGCAGCGGGAGAAGTAATAGAGAGGCCAGCAAGTGTAGTAAAGGAATTAGTAGAAAATGCAATAGATGCTGGAAGTTCAGAGATAGAAATCAAGATAGAAAGTGGTGGGCGTAATCTTATAACTGTAACAGACAATGGAAATGGAATAGAAAAGAACGATCTAGAACTAGCATTTATGCGCCACGCTACTTCAAAATTAAGCGATAGTGAGTTAATAGAAATCAAACATCTTGGCTTTAGAGGAGAAGCTTTGCCTTCAATTGCAGCAGTAAGCAGAATGAAATTATCATCTAAGGCAAGTGGAGCAAAGGAAGCATGGTCTATAAGATATGAGGGAGGAGAAAAAATAAGAGAGATTACCCCTTGTTCTTTGTTGCAAGGTACATATATTGAAGTTCGTGACTTATTTTTTGCCACACCAAATAGACTAAAATTTCTAAAAACCGAAAGGGCAGAAACACAAAGCATTGTTGATATTGTAAATAACTTAGCAATGATTAACTATAGTATTGGGTTTACTCTCACTTCCGGTAATAAAAAGCTCTTAAAATATGCTAAACAAACTTCATTCTTTAACAGACTATGTGAAACAGAAGAAGAATTTCAGAGCAATTCGCTGGAAGTTAAAGAGGAAGAAGAAGGAATCAAACTTACGGGACACATCTGTAAACCAACTATTAGTCGTGGCAATTCAACTCAGATCTATACGTTTGTTAATGGAAGGCCAATAAAAGACAATCTACTTGTTGGTGCAATTAGATATGCGTATCAAGATTTTATTCCAGTTGGAAGGTATCCTTTTGCAGTGCTGCACTTAGAGATACCATATGATCAAGTAGATGTAAATGTGCATCCAAATAAATCAGAAGTAAGATTTCAGAATAAAAGGTTAATATATGAAATAGTGAGAAGAGGGATAATAAAAGCATTATCGACTAGATTCGCAGCAAGTGATGTTAAGTCTCAAAGTATTGAAGAATTTGATGCTAGTAAAAGTCAAGAGAAGGTTGATAGTGAAGAGAAAAAAAATCAAAAAGAGTTTTATGAGAAGAGGCCAAGTCTTTTAGAAAATCGTCTAATGAAAGAATTCAACGCACCAGATGAAAGAAGGCAAAGCTTACCAGAAACGTTTAAATATGGAGAATCTCCACCCCAAAAGGAAGCGATGGTTCTAGAAAGGGAGCAAATTGATTTAATAGAGGATCATCCTCTAGGGTATGCACGCTGTCAGGTCCACAGTACTTACATTATTGCTGAGGCTAAAGGCAAATTAATTATAGTAGATCAGCACGCAGCTCATGAGAGATTGATATACGAGTGCTTAAAGCAAAAATCAAGCATAAAAAGACAAAAACTTCTTCTTCCTGAAATAGTTGAGATTAAAAACCAAGCAGGAATGGAGATGGTTGAAATGTATAAAGATAAGCTTTTCGAAATGGGTTTTGGTGTTGAAATACAATCAGAAGATAAAGTAAGGGTAAAAGAAATACCTGCAATCTTGGGAACAATAGATATAAAAGAGATGCTAATTGAAGTAGTAGATAGATTAACGGAAATAGAAGATACGCTACCAATAGAGGATAAGGTGAACAAAATATCATCCATAATCGCTTGCCACGGGGCAGGAAGAAAAATGAAATTGGAAGAGATGAATGAGATACTAAGACAAATTGAGAAAACCCCATATTCTGATCACGGAAGACCAACGTATATAGAAATGAAACTAAGTGATATAGAAAAATTGTTTGAAAGGAGATGAGTTCAGTTTGTCAAAAAGGTCTCCAATTAAGTATACGTAACAGCTCTTCTGATTATGGGGTATAAAATGTGAAAAAAGAGAATAATTACCCTAACTTTTTAGATTACAAAGTAGTAGGACAGGAAGTAAGAAATCGTAGGTTAGCAAAGGGATATACTCAAAAAGATTTAGCAAAAAAAATCGGAACAACATATCAGGTAATACTGCAATATGAAAAAGGAACACGCAGAATTTCAATTAAGAAGTTATATGAATTAGCAGAAGCATTATCAACAACTGCTAGAGATCTAGCTTGCGGACAAGAAGTATCAAATGAGGAAAGGTATGAGGAAGAAGAGATATTAAATCTAGTAAGAAGACATAAAGAGATTAAGGACCAAGAATTACGTGAAACGTTTTATTTATTAACTAAATTCATCCGTATTAGTGAGGAAGAAAGTGGAAAGACAGTAAAAGTAGAGGTGGCAAAGGGTTTAGTTAAGGAAGGAGTTTCTGCTCATGTTATCTCTCAAACGACCAGTTTATCTATTGATGAATATGATAATGATGAGAAAAAATTTCTATTCCGTATAAAGTAGGTCAAAGAATAAAAGAATGGAGATTGATACGAGGATACACTCAAGAAGATTTAGCAAGCAAAGTGGGCGTAATAAATCAAAGAATATATGAATATGAACAAGGACGAGCTGCTGTTTCACTTGAAATGTTAGATGAAATAGCAAAGATGCTATTAATTAATATTACAGATCTGCTTCCAGAAACAAGAGAAAATGAGAATAGTGAAGTGGAACTATCAAGGTTAATAGAAGAATACAAAAAGATTAAAAGCCAAGAATTACGTCATGTACTAATAAAATCTCTGTTTGAAAGCATACAAGTTTGCAAAGAGAAAGTGAAGAGAGTAGAAAAGATGAAAATTGCAAAGAATTTAGTTAAGGAAGGAATTTCTATCAATATTATTTTAAAAACAGTAGGCGTCTCTTTAGACGAAATTCAACAAATTTAAAATAAAAAATCAGTATATATTAATATCTTTACTTTTTGATGAAAAAATAAGTAAAAAAGATTGAAAAATTGATTTGACTTCACTCAAAAGCTATGGCTTTATGCCAATGCTGACAAAAAAACAGCAGTAAATATTTAAAAACATTTTGTTATTAGTGAAGGGTAATTTTTATGAATAATAAGAAAAGTAGTAGAGAAGAAATAGAGTTCAGAGCATTAGAAAGCAACGGTAAAGCTCTACTTGATCGTGAAGTAATAGAAACGTTTCTAAGTGCAGTGCATAACAGGGAAGAAGCTCGAGTTATTGCTAGAAAGCTAATAGATAATTTTGGAATAGGAGGAGTTTTAGGCCAGGAAATAGATGACTTGAAAACTATAGAAGGGATAACTGACTCTACAGTAGCAGTAATTTTATGCCTAAAGGAAGCTGCAAAGAGAGTACCAAGAGAAGAGTTAAAGAAAGGACCTGTAATGGATAACTTGGAAACCATAGTAAAATATTTAAGGGTGAGTATTGGTTACTCAGAGAAGGAAAAGATGAAAATAATATATTTTGATCAAAAGTGCCGTTTAAAGGGGGAAGAAGTGTTAAATGGAAAAAGCACCGGTATACATAAAGGAGATTATAAAAAAAGCATTAATAAAAAATGCAACATTAGTAATAATGTCACATAACCATCCTGGAGGAAGCTTAGAACCTTCAGAAGAAGATCAAGCAGTAACGAAGAGCTTAGCAGCAGCATGTAGTACTGTAAGCGTTAGATTATTTGACCACATTATCATCACAAGTGGAGGCTATTTCAGCTTTCGAGAAAACGGATTGTTATAACAGAAAGTATTTGCAAATCTACTCATTACAATTTATAATAAGTAATATAGTGTATAACAACATTTGCATATACTTTAATAAATACTTATAATAATAGAGGAGGTTAATATGAATTTAATAATAAAGTAATAAAGTCGGTTGTAGACTTAGTAAAGTATATTTAATAGCGGAGGCTAATATGGTTAATAAAATAATTGTACCTTTTGATGATAAAGAAGGAGGTGTTTACGAATTAAATGTTGATCTTGATAGATTATCAAAAGGTGAGATGTTAAATGCGATTATAGGAATTGGTCGTACTAAGGAGCAATCCACCTTTGTAAGCAAAATTAATAAGGCTAAAAAACCTGGAGAAGTGCCCTTCCCTAATAGAGACGTTCAAATCAACTATGGAAAAGGGCTTGATTATATATATGGTACAAAACCTTTAAATGATCAAGCAGAAGATTTAAATCCATTTGCTTCAAATTTGCAAAAAATAAAGCCAAGTGCAAGCGAAAGTGAAAAGTTGAGCTGGTTTAAGAGTGCTGTGGTAGAGGCAAAAAACATAAATGAATTGCACAAGATTATAGATCAAGCACTAGCTTCTGGAGCAAGGCTAAATGCATGTAATGATGGGGAATGGAGCTTTGCAGAATATGTAGTATTGGGTACACACTTTCACAGATTAGAAAAAAGTGATCGAAAAAAGATAATGCGGAAGCTAATGCTAAGTGGTGCAGAGTTTCATAATACTTTATTGCAGAATAAACTGATAGGTGAAATCTATAATGAGCTACAACCAGAAATTCAGCCACAGATAGATAAGCAACTAGAAGAACTAGAAAAAGCTGGCGAAAGTGCTGTTCAGGAAGGAGAATTAATAGATATTGAGATAGATAATACAACATCGTATATAGAATTTTCTGAGGATAGCAAGGTAGAGGTAGCCAAAATACTGGAAGAATTAGGAAGTAATATTTTAAAAATTGGTAATGATGCAGTTGAGGTTAAAAGTGAGAAAGGAGGTATAAGAAACTATACCGATATGTCAGATGGCAGCTCTATCATGTTAGAATTTCCTACAAGTGCTGGTAAACTAAATATTATATTGTACCAAGACGTAAAAAACTACAATCAAGTACAAGTAAAAGTAGCGGATAAGGAAATGTGGTCTAAATTACAAGAAAGAGGAGAAGAAATAGGAAAAAACTGTCTCTTTGGAGGAGTAAAACTTAAAGAAGTAGTAGAGAGAGGTAGTTTCACTAGATGTGGCATATGGAGTGAAAAACATGCTATAAAAGAGGTTAGCAATAGTGAAGTATTGTCTTCGTGGGTAAATAAGATACGTGAAGGTAGTAAAGAAACTTTTAGAGAACTTTAATGTGTCTACAGCCAATTAAGGTTCCAGAGGTTTTTTCTTACCGTTGTATCTGGAGCCTTAACAAAATTGAGCTATAGTAGAAAATATTATATTTATGGTTCTTTATGTGGAAAAAAGTCTAGACTATGAAGTAGGGGAAAAAGTAAAAAGTTGGAGGTTAGAGAGAGGGTATACTCAGAAGGATTTAGCGGAGAAAATTGGTGTAAAGTACTGGGTGATACTGCAATATGAAAAAGGGAATCGTAGAATTTCAATTGAAAGGTTGTACGCTATGGCGGAAGCATTATCAATCAGTATTACGGATCTTATTCCTGTATCAAAAAGCTGTCTTGAAGATGAGGGAGAAGAGATATTAAATCTAGTAAGAAAATATAAAAAGATTAACGATCAGGAGTTACGTAGGATGTTTTGTTTACTAACCAAATTTGTCCAAGTTAGTGAGAAAAGTAGTAGAAAATCGGAAAAAATAAAAATTGCAAAGGGTCTGGTTAAAGCAGGAATTTCTGTTGATGTTGTTGCAAAAACAATTGGTCTCTCTGCTGATGAATGTGTTGAAGAAAAAACGGGTTCTATCTACTACAAAATAGGGAAAAAGATAAAAGAATGGAGAGTGGTAAGAGAGTACACTCAAAAGGATTTAGCAGAAAAAATGAGTACAACCCGTCATGAAATAAGCAACTATGAGCAAGGAAGGACTGCTGTTCCACTTGATAAATTATATGGAATAGCAAAGGTGTTATCAATTAACATCATGGACCTACTTGAACTAACAGGAGATGAGATAGAAAATGAGCTGCCTGATTTGGTTAAAGAATACAAAGAAATTGAGAGCCAAGAACTACGTAATGCATTAAGAGAGTCTCTGTTTGAAGGTATAAGGATTTGTGAAGAAAAAGTGAGGAAAGCGGAAAGAATCAAAGTTGCAAAGGATTTAGTAAAAGGGGGAATTTCTATTGATATTATTTTGCAAATAATCGGTTTATCCGCTGATCAAATTGCATGAAAATTTGTTTCTAAGTAAGGTATATATGTTTGTTTCTGTAAGAGATGTTAGTTCTATAAATTATAAAATAGGGCAAAAAATAGAAGAGTGTAGATTAATGCAAAGGTATACTCAAGCAGAGTTGGCAAGTAAGATTAGATTAGCGTATCAAGAAGTAAACAGCTATGAACAGGGATATACCGCTATTTCAATTGAAGTATTATATACAATAGCAAAAGAACTATCGGTTAATGTTATAGATCTGCTACCTGAACCAGTAATAGTAAGAGAAGATAAATATGAAGACGAGGAAATACTCTATCTAACGAAAATATACGAGAATCAAAAGTTAGGCAAAATAGTACCTTCATTAGTCAGGTTTGTTCATATTAGCGAAAAAATTAATCAAGAAGAGGCAAGGTTGGAAATAGCAAAAAATCTAGTGAAAGAAGGAGTTTCAGTTGACATAATTTCCCAGGCAACCGGCTTATCTATTTACGAGTATGATAATACAGAGAGAGAAATCTGCACTGATTCTATATACTACAGAATAGGGCAAAGAATAAGAGAATGGAGGCTAATAAGAAGATATACTCAAAAAGATTTGGCGGATAAAGTTGGTGTAACACTCAAGGAAATACACGAGTATGAAATAGGATACACTGCCATATCATTTGACAAATTATATGAAATAGCAGAAGGATTATCAGTGAATATTAAAGTTCTGCTGCCTAAAACAAATGAAGATAATAAAGAAGAAAATAAGCTACTGAGTTTAATCAGAAAAGATGAAAATCAAGAATCACTAGTCAAATCTCTATCTGAAGATATGAAAAGTAGCAAAGAAAAAGTTAAAAAAACAGAGAAAATCAAGGTTGCAAAAAATCTAGTGAAGGCGGGTGTTTCTACTGATGTTATTTTGCGAGCAAGTGGCCTAACTGCTGATGAGTGTGAAAATTGAATTGTGTTAAAGACGGTTGGTAGTGTTAAATATTATATTAAGATATAAGTAAAAATAGTTATATACTTAATATAAATAAAGGTTGCGAATAATCTACGTATGCTGGACGTCTTCTGTTGATGCTATTTCTAAAATAACAGATTTATCTATTGAGGAGCTTGAAAATTATGCTTTCAAACTTAACCTGAATTAAAAGCAACATTAGTTTACTGATTTCTACACAATATATTAAGATATAAGTAGAAATAGTTATATATTTAATATATCTATTCTAAACCCTTATTTAGCTCTGCAGAATTTTTTTGGCATTTCTACGTATCAAGATAAAACATCACAGAATTTATCGACTACTTTTTGATTTTCCTCTATCATTGCCTTTGCTTCTTGCTGAAGAGATTTGATATTTTTTGATGTAATATTATCCATGTCAGGTGATGCTATTTTTAATTGCGATTGTATTCGTATATATTTATCACCTATAACTTGATCCAACTGATAATTTACTGCATCTAAACTTGAAGCAAACATCACATGTAGTAGTGGCTTGATCCATCCTATTTTTCCAAATCTCCTTGAATTGGCTATGCTTCTATCTGTTCTTCCAGTACCTATCGATAACAGTAGAATATCATCATTTGGAAATAACCTCTTACCACTTGCATATGCACAAGCCGCTGGATTATTTGCAAATACTCCGCCATCCACTAATACCATTTCCTTTTGGTTGACTTTTAAATATTTAGGTGCAAAGTAAGTAGGTGCTGCAGCTGCTGCTCTTAATACATCCCTTAATTTAATAAAATTTCTATCCTCTTTCCAGCTTTTGAAGAAAAATGGGCAGTGATTGTGAATATCGTAACTCGTAATCAACACGTTACTTAATGTGTTTTTTAGAATATCCTCTCCAAAATATTTATGAAGTATAAATTCAATATTTTTATGTGGGTATTGTGCGCAATTAAGCCAAGAAAATATTGATCTTCTCAAAAATGAAGATTTAAAAATATATGCTCCGTACTTTTGGTAAAGCTCGACTAAATCATTGGCTGAATATTGGGGATTTCCCCGTTCATCTTTTCTACATAATCCCGCTACAACAATTCCACCGGTTGAAGTACCTGCTATAAGATCAAAGATTTCAGCTACCCTCTTTCTTGTCCTTTGCTCTATTTCTGCTAGTATTATTGCTGGTATTATGCCTCTTATGCCTCCGCCGTCAACGGATAAAATGTATTTAATCAATTGTAAACTTAGGTTAAGATTTAATTTTTCCGCTTAAAACCTGCAATTTAAACTCATCTAATTCTTCAGAAAGATTTTTTACCCTTTCCTCAAGTACTAACACTTTTTCCATTAAACCATATTCAATGAGTTTATCGTGAAATTGTACCCTTGAGTCTAGCTTGGATAACCACCATATCAATGCTACGGTTTGGATTAATAACGTGATAATTACTGTAATTGGGATTTTTTGCTTTTTCATGTGAAAATATTAACTTGTTATGGATAGTTTTAGAAATTTATGTATAGATTATAGAAAACTTACAGCTTTAGTGATTACTTTTTGTTGATTCGTATCGGTCCTCTGCTTCTGCAAGTAGCTTTATTATTTGATCGTAGGGCTTGTCTTTGTTATGCCTTGACCTTAACACAGCTACATTTCTAGGTTTTTTTCTATCGTCGTTTATAATGTTAGGATTAGCGCCTTTTTCTAGCAAAAATTTGATAATCCTTAGATCACCATAATATGCAGCATTATGTAGTGCTGTGCTTCCATACTTGTTAGTAATGTTTACGTTAACCCCCTCATCTATTAAAAACCTAACTATCTCTAAACATCCTCCTTCTGCAGCGTAATGCAACGCAGTTAACTTAGGCATGTGTGTACCAATATCCAATAAATTTTTTACTGAGAATTTTACAATCTCTAAGTTACAATTTTGAGAAGCAATGCGTAATATTGTTTTATAATTATCTGCAGCTTTTGCTTTCATATAGGAATAGGTAGCAATAATACATACTATTACTGCTATAAGCCACCAAAAATGTAGCTTATTTTTTGTATCTTGGCCTAATTTATCCATATTCAACATCTTTTTAATATTAAATAGATCGTTTACTCATTTTTTATTAACATTACTTACTCAAAGACGCTGACAACATAGTAAGGTAACCCGAGCTATTTAAAGTATGCTCCGCTCTATTAACTATCCATTCACCATCTACTGCTTGATTAAAACCTATAAGACTAAGTTTAGCTTCTGCAAATAACTCCGGATTACCAGGCATAGTTATATCTAAAGTTTCATTGTTACGCTTCAATTGTTTTAATTTGGCATTTGCTGCACTTAGTGCTGACTCTGCATTTGAGTAAAGTTCCAGCATAATATAACTTGGTTCACCGCTACCAACTGTTTCTTTAATAGTTTTGCCCTTTTCATAGCTATGCCATTTTGCTACTACTGAATTATACTTATCACGTACGGTAAAATGCACTTTCCAATTAATTGTGTCTTGAGGTCTAACAGTCGTTGTTCCTAAAGCTTTTCCTGTAGCTGATTTTGCCATGTTTTTTGAAATAAATAATATATACCCACCAGCTAATTTTACCATTGCTTCACGCCCTATTGCTATTTTCGTTAGAAGGCTTATATCACTCTCCTCAGTTTGATTAATGTGAGTTATAAGTACGTTTTTAAATTCCTCAGCAACTTTATGTCTATATCCATGTTTTTGGGCTATTTCTTTTACTAAATTTTCTATGGTAATTTGGTGCCATTCTTTTGATACTTTTGCTTTCAAAGATATTCTTAAATTTGTTGCATGAGCTTTGATTAGTAGAGTCTTAGGTGGGCCTTGTATCGTAACTTCGTTGACTGTATATATACCCATTGGAAAGATTCCCGTTTCCTTATAACCTAATGCTATGTTCAATTCATTTGGAACTTCTACATTTTCATTGCCATAATCAACGCATACCTCTGCGACATCATCTATAGTACCAGATTCATCAGTAAGATGCACCGATATTACAGGATCTTTTATTCCTTCAATGCTAAATTCAGGTTTCATTTACTCCCACACTTTTAACTTCGATTTTTTTAATTGCTCTTGTATTATAGGTAACTTAATTTTTAATCCTGCAGGCAAAAAACTTCCATAATCTGCAATTCCAGGATTTTCCTCCAATACTATCTCTACTGCTCCAGAGCTATATCCATAATGTTTCCAGCAAATTTGATCTAACATTTCATTTTTTCGGGTTATATAATGTACTGTCATACATAACGCCTTAAACTTAAGCTAAATTCAACCTTCTTTGGTAATCCACATGGAAAAAATGATGTTTGTTTTTTCTCTATCCGTACAATCACAAACCTCCCTAAAACATTACCTAAACTGTCTACTAAAGTATGTGGTTCTTCTGCTTCTTTCATACTTTTTAATTGATTTAAATCATTGAGATTATGAAAATAAATTATTCCTTCTAAGTCTATATTTTCTGTACCTTGGCCAATATTTTGTAATGAAGGTATTTTACCAATACACTCAATAGTACTCCAACGATTTTCTTTACTATACCTTACACTTGTTGGAGAAAGCTTATGTTGACCAAACGATAGCATTAGTAAATCGGTTCTATTGAATCAAATAGAACGTCACGCGATTTTTCTCTTATTCTTTTTATTACTGCATCGGCAAGACTACGTACATCTTGATTAGGTTCTGCTTTTATATTTATATTAAATGTAAAAGTTTGATTGAAAATTTTTTGTTCACACTTTTCACAGTCTTTTAAAGCTTTCTCTGAATTATCTACTACAAAACTTTTTTCTGTAATCGCACTAGAACTTTCAATCATATTCGTTCTACTATTCGAAATTCCACTAAAAGTATTATTGTGCAGTAAAGGATTTCCTTTGCTAAAAACATTATTTTTACTGATTATCTTAGTTGTACCGCCTATTTTTGCTTTTACTGGTTTCTCTAATGCTTTTATTGGACTATCATTAAACAATTTTCCTAAACCAATCCAATTTTCTATAGGTTTTGCAATTGATTTCCAGATACTTGAGAAAAAGTCCTTTACCTTTTGCCAATTAGCGATTACAAGTGCTGCACCAACTGATAGTCCAGCAATAGCAGCTCCTATAGGATTGGTAGCTACAGCTAGCGTTAAAGCTTTCAGTCCCATGATTACTGCTGGAATTACTCGAGCTGATAACGAAGTTAATACTGGCAAAAGTGTTCCATGCAAAATAGCCTTAAAAGTTAATATTCCACCTCCAGCCAATGCAAATGCATAACCAAAACCTACCACTGCAATCTTGCCAATAATAAGAGCTGAAATTATGCTCATAACTCCTGTAGTCAAAATTGGACACTTCTCTGCGAACCAAGCTATACCTGTAGATATAGACCTCAAAATCTTACTTATCCAATTTAAAGGAGGTAGCACAACTGACCCTAAGTTCATTCCTAGCTCTGCTATTGTATTTTTGAGTAGCTGCAAATTATTTGCTGTAGTACTTGCGCGATTGTTAAATTCTTCTTGCATGGAATATTTATACTTTTCTTTGTCAGCTACAAAAGCTATGGCATCTTCATATTTTTTTAGGCTTCCAACTATCAATGCAATATCATCTTGATATTCTTGACCAAAGAGATTGAGAAGGGTTTGTGAACGCTCCTGTTTATCTATTTTCTCTAAAGTTTTAAAAAAGTAGAGTAATGCTTCTTGGCCGTTTTGAGCAATTTTTTGTGACATCTCTTCTGCAGTTATGCCCATGGATTCCAATGTTGCTTTAAATTCTCTTCCTTGTCCTTCAGCAGTTTGAAGTTTGCTAAGTAGAGCATTTATAGCAGTTGCAGCTTTTGCTGGTTGTTTACCTAAACTAACGAATGCATTTACTAAACTACTTGTTTCCTTGATATCAAAACCAAATTGTTTTGCAGTACCACCAACTATTGCTAGAGCTTCAACCATATCTTTTGCTTTGGCAGCAGTGTTATCTGATAAGTGGTTAATTACATTACCAACGTGTTCCATTTTACTAACATCAATTCCATAAACGTTAGAAAGTTTAGCTATGGAGTCACCAGCTTGTTCAGCAGACATGTCAAATGCTGTGGCCATTTTAGCTACTGTTGTGGTAAATTCAAGTAGCTTACCTTTATCGATACCAAGCTGACCACCACTTGCAGCTATTTGTGCTAACTCTGCGGCTGATAATGGTATTTCACGAGATAACTTCTTTAATTTTTGAGCAAATTCAGTAGCTTCGTTTGTCCCTTCTTGAAAGTCTACTACTTTCTTAACATCAGCCATAGCACTTTCAAAGTCAATCGCAACTTTAATTGGCGCTGCAAGTGAAAGCCCTAGTCCTATAGTCTCCATTACTTGTGACCTATAATGCGCTTTTTTTGCCAAAGCATTTTGCTGTTTTTGTATTACAGATCCTAATTTACTGTATTTTCCTTTAAGTACTTCAATAGATGAACCAAGTTTAGTTTGATCTCTCACTAATGATTTAATATCCTTTCCACTTTTCCTTACTTCTTCATTTAATGTGTGAAGTGCATCTCTCTTTTTAATGTAAGCCTCTTTTGCCTTTGATGCCGATGCTTTCAATTTTTCAAATTCATTTTTCAGTGCTTTGCTTGGTTCTTTTTTTTCCTTCTTTTCTTTAGCAATTGCTGCAGCTGATTCTTTTGCTTTCTTCTCCGCTTCCTTCCAATTCTTCATGGCTTCAAGGGCATCATGATTTAGTTGCTTAAATTTGGAAACAGATTTCATTGACGAATCAAGCTGCTTTATACTATCACCCAACTTAGAAAGCTTTGCTGCACTACCTGTCATTGCATTATTAAAACTGCCATCTAACGTTGCACCTATTTTTATTGAAAGCATTGACATTTTTTTGTTACTCCTTCACTTACTTCTAACCACTTTAGAAACTCTTTTATACTCATATTAATAATTTGCTCAATTCCACTTCCTGTTGTTGAGCTAAGTTTTAAAATATTGAATCTTAACTCCTCTGTTCCAGCGGCGACAAAAAATCTTTTAGCACCTTCTGTATTTCTACATAATCTTTAATACATAAATCTTCAACTACTTCTTTTGGTACGGATGCTAGATTAGCAATTAAAGCTACTTCTTTTAAAGCTTCGCCTTCTATGCGTTCTATAGCAAGTAAATCTCTTACTTTAGGTTCACGCATCGATAATTCTGAGACAGAAATTCCATCAACTGTAATTGGGTTGTTTAGTGTTATAGCTTGCATAAAATTCTCCTATAAAAAATAAAATCTTGTAAAATGTTGTAACTATTTGATCAACATCTAAAAAATAGAATTCTAAAAATCTTCATATTCCAAGCACCGTTTGAAGTAGTGCCATTTGATCAACACCATTTATCTTTCTAATCATATTTTCAGCATCGATTTCTATTAGCTCATTACCACCTATAGTAAGTTTATAGTAATGGGCAGCTACAGTACACTTTAGCGTTGCTTTTTCAGCAGGTTTCCAGCTACCAAAGTCAAATTCTTTGAATATTCCCCTGAGATTGATTACTACAGCTTCAATATCGTTACTACCACTACCTTGCATTCCACCACGGAGCGTCAAAGCTACTGAATTTCCATTTATCAACCCAAATAGCCGAAAGAGTTCTGTATCGTATTCAGAAAAAGTAAAATCTGCTTCAAGCTTTTCCATGCCCATATCAATATTTATTGGAATATCCATACCACCAGCACGGTATTCTTCTGTTTTTATGGTGAGCTTTGGCAAGGTTATTTCATCTATTTTTCCTGCATAACCCCGACCATCTACGAATACATTAAAATTCTTTAAAATCTTCGGTAACATCTCTTTCTCCCATTTTTATAATATTGTACCACTCACAAGATGTGACCTGAAAGTAATCTGTTCTGCTGGATATGGTGGTGTAAATTCGAAATCAAAATATACTTTTCCGCTTGCAATGTTTGCCGGTGTATTGAGTTCTGGAGTTGCGTAACATTTTCCGCTGATAATTGCTCCTTGGGCTTTTAAATTGGCCAAATAAGAATTCACCCCCTCAATCACATCATCTATATAAGTTTTGGTGATATTTCGATCAACTGCCCATAAATGAGCTCGAAGTAGACTATCGTTGATTAAATCTGCAGTCCTTCTCACTGACAAAAAAGCCCATTTTGAGTCATTTGAACATGTTCTATTTCCCCAAAGCCTATAGCCATTTTGATGAATTATCGTTGTTACTTCATTTTCATTTAAGTGGTTTGCTCTACAATTTGTATTACCGAGCGTAAAATCAATAGGCCTGCTTGTTCCAACAATACCATTTATCTCTTTATTTGAAGGTGAGTGCCAGAATCCTTGTTCGCTATCTACTTTCGCTATTAAACCAGCTACAAATGGGCTTGCTGGCAAAATTTCTTCTTTTCCTTCAATAAATGGATCAACTACGTAAACTCTTGAGCTACCTACACTTTTTCTCCACTTTATTGCTTCTTCATCATTGGTATTTGGTCCATCTGCTACTATTATTGCTCTTAGCTTTTCTGCTATAGGAATTAAAGCGCTAACCACTGGATTTCCAGCATCTCCAGATAATTGATGAGTAAACTGAGGTGCAATTAGTATTCTTGGCGCAACATGAACTATGCTTTCACTGCTTAAGAACGCTTGAATCCCTTGATACTCTCCAGTTTCTTCATCAACTCCACCAATGATATTGCTGAGCGTCTCTTCTACTTTCTCCTCAACTCGAATAACTACTACTGTTGCACCAATTTGGGAAAAAATTGCATTAATAGCTGAAGGCAAAGTCCCAGATTGACCAAGTTTTGCTGCTTCTTTTAAGCTTCCTGCTATTAATACTGGTTTATTTAGTGGAAATTTTTGCCCATCAGCTTCAGGTGCAGTACCAATTACTCCTATTACTGATGATTTAGCTGTACGTACTGTCCTTGCCCCTGAGGTTACCTCAATAACATTTACTCCGTGTAAAAATTCTTCAGCCATTTCTTTGCATCTTTTGTTTAAAATCGTTTAGTAAGTTCTCTAGCTCTTCTTCACTTTTGGCTTCTTCAATTTTCTTCTTGGCGATGTCTTCCAACTCTTCACATTTAATTATTGCTTTTACTGCTTTTTTACTTTTTCCTCAATTATTCTTGCCATTTCAATAACTGTAATACCACGGACTTTTGCTAATGGCTCTATAATTTCTGCATCCTTTTCATTCATAGATTCTGGTGCTGCTAGAATGCTTTTTGCGGCTTTTTCTTGTATTTGATACGATTTAGCTTTTGGTGAGAATATCCTGCGTATTGGTGAGTATAGTTATCATAATAAGCACGAAGATTAGAAAGTGCAGAAAACTTCGCATTTTGCAATAATTCCAATTCAATATCTTCTTGATTTCGCTGAGCAATTTTGCCTCCTTCTGTTAAACAATAGCTTTTTTGCCAATCAAAATCTTTTGGCGCTTCATACCAATCATTTCCAGTTGGCTTGTTTTCAAGTGTTGTTGTTTCAATCTGCTTGTGGTTTTCAAAACGTATATAAATAGTCACAAATTCCTCCTCATCGCCATATTTGATCAGTTCGTTCTAATCCTGGACATTGCCAAGCTCTTAGCGTTCTCTCTACGTCCACCTCAAGTCCTGTAGTTAGAAAATTGCTACGTATGTTATAAATTCCCCACTGAATAAATTGACCATAGATTGTGCTATACGTGTGCTCACTTGCAAAGAACTCAGGTGTCTCACCAACTCTTTCACCTTCTGAGTGAAGATTGGATGACGTATAAAGTAACACTGTAACAGTTTTTCCTGCTGGAATGATTATGTTACCTGATCCAATAAATTCAGAACTGCTAATTTCATGCTGATAAATACGTGTCCAAGTAATTCTCGAAATCTCTGACTTCCTTGTATTAGTTTTATCAGGTGTACCTATAAATAATCCTGCTCCTCCATAATTAACTTCATCTTTATACCACATTGCAGATCCAACAAAGTTTATAGTTTTACTTATATCTACATTGCTTGTGTTTTTTACGAATATTACTCCCAATGCAGCATAAGGGTATTTCAGCATATTGAATTGCTCAGAAAATCCATCATACATGAATCTTGGGTATGATTCTCCATAGATAAATGTACCTTTGCTTCCCTCTAAAAAGCACAATTGTCTTGGCCGATAAAAACTAACATAGCTTGTATCATATGTGTGAGTACCTGCTAGTAGTTGTAGCATATAGTCTGTTTTTGTTATATCACTACTCCACTGACCAAGCTCAGTTGTAAAAGTTCCGTGGCCAAAGTAATTATTTTTTCTACTTAGTACACCAAATAAAAAAGGTAATGCTCCAGGTTCAATCATATGACGCTTTCTTACTTCTTTCATGATTGATGAGCCGCTTGGTACATCATTTATAGAGTTAAGCTTAGTTGTTAGTTGATTTTCTCTGCTCCTTATCTCCCTCATAATCGATGAACCGCTTGGCACATCATTAACAGTACGGAAGTTATTCACTAAACCTTTTAGACCATCTTTATGATCATTGCCAACACTGTTTATTGCTGCAATATTGGCATTCTTTCTCGTATCAAGTAGTGATAAATTTGTTGTTCCTTTTTCATCAATTCTCTTTAATAACTGCTTCTCTGATTCAGAGATCACTGCTAAAGAACTAGCTTTTTTATTATCAAGATCAGTTAAGTGTTTTTTTGCACTATCAAGAAGTTCTTTTAGCTTACCATCAGTCATCTGAACAATTTCAGAAATAGCACTTTTGTCAACTATTGATTCCAATGCCTTAGCAAGATATGCAAGCTGATCTGGAGTACTATTTACCGCTAAATCCTTAAGTCTTTTTTGTAGTGTATCAATTATCCCTTTTACTTCAGTCATCTGCACAACGTTGGAAATAGCTTTTTTATCTGCTATCAACTCCAGCGATTTTGCAAGATATGCCAGTTGATCAGGTGTGCTGTTTGCTGCTAAATCCTTTATCCTTTGGTATATTGCTTCTTTCATTCCCAAAAATTCAAAAAAGTTTCAAATCTAAATCGTTTAAAATTATTTTTTAGTTGATTATGTTTATCTTCTCTTTCGGTAATATCTTCATCTATTTTTTCTATAGCTCTTCGAATACGTACTACATCTTCCACTGCAATGTTTTCTGGATGTGGTAGTGTATATCCTCGTTTGCTTTGGTTATTTGGCATTGATTCAAGTAATAATCACTCGCAAATTTTTGACTTTAGGACGATAAATAACCGTTCCACTTAAAACTAATTTTATCCTTGTCTCATTGCCATTAAAGTTTGATAGCACATGAGTTCTCTCCACCCAATTTTCTCCAATTGGTTTTCCTGATGTTAAATTTACTAATTGCCATTCCACGTTTTTTTGCACATATGCTTTAACATCTGCAGTGCCAGGTATTAGCGCATCATAGGTTATGGTAATCTTAGTGTTAGCTCCTGCTGTAATGCTTCTTGTAACATAATCTCCAGACTCTGAAAGATTACCCATAACTAGCTGTAACCCAGGATAGAGAACTGGACTTTTTCTTTTGACCCTTTTAAATTTGCCTTTACTGTTAGCTCTCCAGATAATCTTTCACGCAGTGCAAGCGGCAAATTATCAGACAAAAAGTTTTCTTTTCCCTCTTCATCTGTTAAAATAAATTCTACATTGGTATCAAATGCTACTTTTTCGACGTTTGTCAAAACTATTAAATCTGATACGTTATTTGCAGTAACTTTGCCAAGATCAATAACATGAGAATTTTCGCTAAATTTTGCAGCTAGTAATCGAAACGTTAAATCTAAATTTTGATGTGGTGTCCAGGTACTTGCATTGCTTGATGACAGTAATACTCCTACTTGATATGGTTGACTTGTTACCCAACGGCTATTTACTGCATCATATTTGCCAAGTTCTGCTATTTTTACTGCAGTATCTGCATCATCAGTAAGTAGCACTATTGCATACTCTTGTCCTGCATGGCAAAACACTGGTGACCAAGTAATACGTGTTGCTGTGCCATCTATCTTTATATCTTTTGGCTCAATATAGCTTTCAGCAATGACAGTCTGCGAGGGCATTCCCACTGCTGTTTCTCTAATCTGCACAACAACACGTTTTTTGCCTTTATTTACAAACCATAGCTCCACACCTCCTATGTGTCTGCTTTCATTTAGAGTAAATGTTTGCGCTAAAGGATCAACTCGTCTTGCTGCGATAACTCTTCTTCTTTCTTCTATGGTAATAGTTTTTTTACCAGTATAAGTTGCTTCTCCATAGCTTCCTTTATCCCCATAAAACTGTACTAATTTAGTACCTGCTGGAATATCTGCTGGGATTTTTATTTTTCCACTTACTCGCCCTTGATTATTAGCTGTTAACATCTTTTTTTACGCTGTAGGTTGAATAATTATTCCGTCAAATTTTATCTCTTTAAGCTTCTCATTTGGCTCAAAACCTTCGATCTCAAAATCTTGTACTGCTTCTCTCATAAATTCAGCTTCATATGAATTACTTGACAATAACTCTGTTGTTTCTCTAGTATTAAATTCTCTTGTTACTGGACTTGACCAATTGGTCTTTATCTCGGTCCAGTGATCGGTGTTTTTGTTCATGGTAACTTTTGCCGGTACTGGATCAAATGCCTGATATGGATTGATCTTTTCACCAGTCTGTAAAAGCTGCTCCAATACTGGCTCAAGTTCATACGGCAATAAATGAGGTTCTTTTCCCTTCTCAACATCAATAATTTCCACGTTTATCGGCAATATCAGTTCCTTATTTACTATTGCTGCAGTTTGGGAAATACCCTGATCACGCATATCATCGTCAAAGAACGGATCAACAAATACTCCTTTTTTCGTGGTTGGCTCTCTTGAATTTGCATCACTACGTAGACGCTCCTCTGCAACTAGCGCATAAAGATCTCTTATTCCACTCTTCATTGCTTCGAGCTCATTCATCGGCACAGCATGAATAGCATTATTCATTACTTTCACCCCTTCTTTTTCCCCGGTTTTCCATGTTTGGTGAATGTAGCAGAGCAAAAGTTGTCCACTAGGCGCTTTAGGCATTGATGGTCTCCAGGGATGGGCAATTCCCTTGATTCTTCTTACCACTCCTTTTGCATCTATAGTAATTAAATCAAAACGGGGCATTTTCCAGGTGTAATCAATCAGAACCAAGCTGTTATCAACTGCACCTCTTACTTTACATCCCTCTTCACTTATATCTTCAGGGCTTACCTGAGTGCGACAGCGATAGGTTATTAGGTAACTACTTCCAGGAGCTGGTTCTTTGCCTGGTAATGACCAATCAACGTTTCCTGCGTTTAGTTTATAATCTATACTATTTTCATAAATAACATTGCCTTGTTTAATTTGAATAATCTCAAGTACTGCAGAGTCAGGTATCGGATCAATAGCTCCAGAATATGAACCATGAGTAATGGTAATGGTTTTTTGAACAGTTATATCTACTTTTTTAATTTCACTTATTGGAAAATCATTAACTTTCAGTTCCATTACTCTTTGGCTATTTGGCTGAAAAGTATGTGGTTCTGATTCAACTGATTTTATATCTGGATCTTCATCAAAAGAAACACGAATACTGTGAGGAAGTTCAATCTCATAGCCATCAACATGAGCTTTGCCCTCATTAATCACAAATATTTTTTTTCCTCCTTCTCCCTCTTCCTTTTGCAGGAACATTACTTCAAGACCATTTACGACGTAGGAACCATTTGCTTCTTTGTCATAACGAGCAAGAGCAGTAGTTACTATATTTGCTTGTGGTGGCGGTGAATGTTCTATCAATACTCCATTTTCAATGTTATAAATTGGATAAAATTCTCCTTCAGAAAAACGTGGAGAAAGACCTTCTGCTTGATAACCCCAAATGGTGGAAACTTTAAGCCTTGCAGCTCCTACTTCCTGATAATTACGTGTACCAACAGCAGGATCACGAAGATTTTCATCCTCAAGTTCTGTAATCGTAGATTCTACATAATAAACACCTATACGCACTGTGGTACTCAGTGGAATAACAAATTCTTCTTTTTCAACTTTTCTAACTGCTCCACGAAGATAGATTTTTCCTGCTTCAAGTGTAACTTTACCAGTTTCTCTATCTATAATACAATTGCTTCCTGTTATAACATCACCATCACGAAATATTGCATCACCTATGCCTTTAAGCTTAGAGAGAGCATACTCCTGAGTCTCGTTTAATTCTGCAGATTGCAAACCTCTTCCAGCAAGAAACAAGCTTTTTTCGTATTCTTTGTCAGGATTAAAGCGGTTATAATAACTATTTAAGGTCATTTTGTTTCAAAAAGTTACAACAAATGAAAAAGTTTCCCGAGTTGCTGCAGTTCTGATAAGTGGTACAGTGTGTTCTAAAACTAACAAAATTCCCGGATCTTCTACATCTTTTGGTTCAAAATATCTCTGTCCAGGAGGTACTTTTTCCTTTACTTTAGTACCAACCATAACCCCTAGTTCCCGTATAACTTGATTTGCTGCGTCCGTGAAATCGAAAGTAAATTTGAGAAAAAGATTATTGGTTGGTACATTAGAAGGCCTAAACCTTCCAGAAGGAGTTATAAGTTCGCCGTTTTCATCACCTGTGCAGAATAGCACTTCATCTGCAGTACGTCTGCCAAGTTCATTGAGCAGCTTTTCAGAAGTTATCAGCTCTGGGCTGGTGTATTTTGAGTATACTCTACGGTAACTGTGCTACCTGCTGCAATAGAGCTATTTTCCGTACGTTTTATTATACCACTACTGCTCTCAACTATATAGTCAATACTGGATTGATACACTGTTTGTCCTGTAAAAACCCTTACATCTTTAATAGTGTGGTGATCTAGAGCTATTTCACCCTCAATAAAAACTTTTTCTACCTTATGGCTACTTTCCCAGCTTGTATCACCTGTTCCCCAAGCAAGATGTATAGATTGCTCTTTTATACTTGCTGCTATTGCTGCTCGACCTGATTGTGTAAGAATTGACACCTGTGATGAGTTTGAAAGGCTTATATAATATATATTCAGGATTTGGAAAATTTTTGTCCAAAAAAAATGCATTTTTGCCTAATTTTTTATTGCAGTATTATTTGAAAATCGAACTGTATTATTCTCGAAAAAAACAATAGAATATCAATAGGTTAAAAATCGCATCTGTATTATTTATTTTTGCTCCTGTCCTACAAAGTGCACCTATAAATTCTCATCCTTAAAGCTATTCATTGTTTTAGGGAGAAGTTATGAAACGTTATTCTGGTGTTGATCCTATAGTTGTTCAAAACATACAGTACCAAGTTAAAAGGTTAAAATTTTTTGAATGCTTTGCTCATGAAACTCATGAAGATCTTGAGCAAGAACTCTTCTGTGAAATTTGGACTTATCTTGATAGATATGATGAAAGTAAAGGTAGCTTTAACACTTTTGTAGCAAGATTAACTAAACGTCGTGCTAACAACTTATTAGAGAAACAACTATGTATAAAACGCAATATTAATAACTACATCAATATTGAGAAAATAGAAGCTTTTGAAGATGAAGTAGCGAAACGTACTGATGTAGATTACATGATTTCAACGCTACCAAGAAAAATGCAAAAGATATGCGAACAACTCAAGTACTTTAACTTATATGAAGTTGCTAAGATGAACAACATATCAAGAACTACTTTAAATACTATGATAAGAAAAATACGTACAAAACTCTCTTCCATCTACTACAAGGGCAAAAAGAAAAATTGAACATAAATTCTGCCTTTCCTGAATATATAACATATAACATTGAGGTGAATAAATGACTTTTAAAATTTTAAACAATAATGAAAGACTGAAAACAACCACAGGCATAAAAGTAGTAATTTTTGGTCCTTACGGTATTGGTAAAACCAGCCTCTTAAAGACTATAAGTGAACCAACACTTTGCCTTGACTTTGAAGCAGGTCTGCTTGCTGTTCAAGATTGGCAGGGAGATTCAATTAGTGTTCGTACTTGGAATCAAGCTCGTGATATTGCCTGCCTAATTGGTGGTCCGAACCCTGCACTAAAATCTGATCAAGCGTATAGCCAAAAACACTATGAGCACGTATCAAGTAAATACAATGAAGAGTTTTCTAAATATCGATGTATCTTTATTGATAGCATCACTGTAGCATCACGTCTTTGTCTTTTATGGGCAAGAATGCAACCTGAGTGTTTTTCTGATAGATCGGGAAAAGAAGACAAAAGAGCTGCTTACGGATTACTTGCTCAAGAGATGATGGCTTGGCTCAATCAATTTCAACATATCAGAGACAAAGACATCATCATAGTTGGCACATTAGGTCAATATCTTGATGACTTCAATCGTCCAACCTGGCTGCCTCAATGTGAAGGGGCTAAAACTGCTAGTGAAATTCCTGGGATAGTTGATGAAGTAATCAGCATGGTTGGAATCAAGAAAGATGATGGCACAGAGAAACGTTCATTTGTCTGTCAGACTATTAATACTTGGGGATACCCTGCTAAAGATCGAAGTGGCTGCCTTGATATGGTTGAAGAACCGCATCTGGGTAAATTACTTGCGAAAATTAAAGCCAAAACTTTGGCTCCTACTGTTTAATTAAAAATTGGAGAAATTATTATGGAACAAAGCTTTTTCAACATCGGTCAAAAAATTCCTTTTTTCAGCGTAAAGGAGTATTTAAGTGATCAAACGCCAATACCAGAAGATATAATCTGTCCTAGAATTTTAACGAAAAGAGGTTTATTGGTACTAGGTGGCCCACCTAAAATCGGCAAAAGTGACTTTTTGATCTCTTGGCTTCTCCACATGGCTGCTGGTAGATCATTTCTTGGTATGACACCAAATAGACCTTTGAAAATTTTCTACATGCAAACTGAAATTGAATATGAATATATGAAAGAACGGTTGCAACAACTTGATAATGAACTTTTGAATGTAGCTGCTAACAACTTAATCATTACCCCAAAAGTGCACTTATCATTTAATCATGATGAAATAAGTGAAATTAAGAAAATTGTGAATGAACGCTTTAAACCTGATATTATTGCGATTGATCCTCTTCGTAACATTTTTAACTCAAGTGAATATGGCAATGAAAACGACAATAGCGCTATGCTATTCTTTTTGCAAAAAACACTTGAAAGACTGAGAAATGTTATTAACCCAAATTCGGGCATAATACTAACCCACCATACAAAAAAACTATCCAAGAAAATGTTAGAAGAAGATCCATTTCAGGGTTTGAGCGGAGCTGGTTCTTTGAGGGGATTTTATAGTACTGGTATGGTGATGTTTGCTCATGATGAGGAGAGTATTGTACGTCAGATAGTATTTGAATTGCGTAATGGTGAACGTGTGGCAAGCAAGCTTGTCGATAAGATAAATGGTCGTTGGAAACTTGTAGACCAATGGAGTTGATTCTTTTTGCAATTAATTTATAGGAGGACAATATGCTATCAGATTTTTTAACTGATTTTAATACTGCAAAATCACAAAGTAATTTAATACCAAAAGGTACAACGGTAAGAGTCAAAATGGCTATTAAACCTGGTGGTTATGAAGATTGGTTCACTAAAAGCTACACTACTGGCAGCATCTATTTAAACGCTGAATTTACTGTCACTGAAGGGCCATATGCAAAACGTAAGATTTATCAAGTAATTGGTATTAAAAGTGGCAAAGCAAGCGTTGAAAAAGAAGACATCTGGGGAGAATCTGGTCGTTCTATGCTTAGAAGCATTTTGGAGTCAGCACGAAATATTCATGCACATGACACTTCAGAAAAAGCAATTATTGCTAGAAAAATCAACTCTATAGCTGATTTTAACGGGTTAGAATTTACTGCAAAAGTTGGTGTTGAAGCTGATCAATATGGAGAAAAGAACAAGATTGCTACTGTTATTACTCCAGAACAAAACAATAGCATTGAATCTGATTGGGTCCCATTTTGAAGTACGACGAGGAGAAGCTCTGGATTGCTGTTATTGAGAGAGCTATGAAAGATGCAGCAGGAAAAAACGTAAAGCTGAAAGGAGAAGCGATAAAATGGTTAAATTCAGAGTCTTTTGAAACTGTTTGTGAGCTAGCTAATCTCGATTTTAAACTTGTGAGAGATAGCTTATCTAAAGAACTAAAGGGTCATGAGAAGGTAAAGCTTATGAGTGCTTAAAAAATTTTTCCACTTTTTTCATAAAAACCCGTGGGAAACACGGTATATATATAGTAGAGGGGTCAAACTTTAAATGTCAATTCTCACACAATCAGGTCGAGCAGCAATAGCAGCAAGCATAAAAGAGCAACCAATACATCTTGCCTGGGGCAGTGGTGATGCCAATTGGGAAAGTAGCCATCAGGTCGAAAAAGTTTTTGTTGAGGGTGAAATCAAGCTTGAACACTACCCCATTAAAGATATAAGGGTTTTTACAGGACAGACAATTTATCAGTCAAGTATAGACTATACAGTTGCTGGTGTAATTAAACGTACGGAAAATAGTTCTATTGCAGAAGATGGTGCAGTTACCATAGAGTATACTCAAGATACACCACCAGAGTCCATTACGTCTGAAAAACTGCTAAATGAACTTGGCAGACGCGTTGTCGATGAGGTTCTCTTCTGCACAGGTGATGAGAATGGAGAGCTTGTAACTCCCTCTGGAAGGTTTAGGCCCTCTAATGTACCAACCAATAACCTTTTCCTCACATTTACTTTCGATTTCACGGACGCAGCAAATCAAGTAATACGGGAACTAGGGGTTATGGTTGGTACTAAGGTAAAAGAAGAATTGCCTCCAGGACAGAGATACTTTGAGCCAAAAGACGTAGAAAATCCGGGAATTTTGTTAGTTTTAGAACACACGGTACCACTTATCAGGACTGCGGCAACCCGGGAAACATTTGCCTTTGTAGTAACATTTTAAAAAATTTATGAAAGGAGAAAAAGAAATGACAAAAGATGCACTTACTCGTTTGCAGAAGATAGGACTTGATGATTCCACTGCTGAGCAGCTACTCAGTTTTAAAGATAACGGAAATCTTCTATATGACTCAGCATTTAAGATTACTGACTTATCTGAAGATAGGTTTGTTGAAGCAGTAAAAGATACATTTTTAGAAAGCACGGCACGGGATATCTACAAAAAAGCTGTAGCTAGGAAGAAGTTTATCTCAGTTTATGTAGCAAATGCTAAGGAAATTTCTGAGCCTCATTATCGAGCTCAACACTGCTGCAACATACATTAAAATTTATAGGGGAAAACATGCATATACCAAAAATCTTTAATACCGATAATTACAATCAGTGCGAAAGTTGCCAATCAGTACTAAGTCCCGCAGCTTATCTGGTTAAGTTATTGGAAATAGTTGATAAATATATTACACAACCAACTAAATCACTTAAGGAACGTCGTCCTGATCTCTACGATATTAAACTCGATTGCGACAATACTAACAAAGAAAAGTTATATCTTGAAATTGTCAATGAGATAATGGAGAAAAAGCTAAAACATGATCTTGGAGATGATGTTCTACGCAAGCTTGCAACTGCCAAATATCCTTTTAATCTTCCGGCAAATTTTCCGCTAATGAGTATACGTGCTTACCTTAAGAAACATAAGACGAGCTTAGCAGAGATATATAAAATGCTCATTGAAAATGCTGACACTGATGCAGAGTTTTTAGGTCTTTCTCCGGAAGAATATAAAGTAATTACATCTGATAATGAAACAGAAGCTTATCTAAAAGAAGTATACGGAATAACAGAAATAGCTCAATTAAAAAATGTTGATACATTCATTGCACAAACAGGTATAGAACACGATAAGCTTCAACCATTGCTTGATAGCTATAATAAAATAAAAGGTTCAATAATACTTGCCGTCACAGAACAAACTATAGATGAGCACAGAAAACGAATTATAGATAACCTTGATAACCAAGCACTAAGCTTTCTACATCGCTTTATTCGTTTAGCAAACAAACTTAATTGGTCATTTGACGAGTTAACTCAAGCACTAAGTGAGGGTAAAATTGATCAGAAAAAAATAGCAAAGATTAAAAGTCTACAAGAAAAGTTCCAACAACCTTTAGCTAAAATATTCGCATTGTATGCTGATAGCTTACCAGAGAGCGTATCAAATAAAATTGATAATTTAACAGAATTTAAAACACTTTTAAGTCAGAAAAGCCTAAAAGATCTAGCTGCCACAGCAAGTAATTATAGCGCATTGCAAAATATTTTAGGAGTAAACAGTAATGAATTAACTTCTTTGATTAACTACCATAACGATCAAAAAGTATTTGGGGAAGAGATTTTAAAAGTATACAAACAAGTTCTGCTTGCAAGATTGATAAATATACCAATAGCAGAGCTCTTATCACAATTAAAGATAGAAGGAGTAAATCAAGAGAACGTAGTTGAATTTAACGATTGGCTAAAGAAACATACAATTACCACTGAACAAGTAATCAGATTAATTAATCCTGCTTTAAGCAAGCAACTTGCAGAACAATTGCACAATTCAGTACAGGAAGTAAAAGATCTGCAGAGTTTCTATAATGAAGTATATAAGTGCATAGGCAACATTCAACCTGAGATACTCGATGCTATTTATACGTTTACTCCTCACTCGGAAAATCCACGACCATTTGCAGAGAAAGGATTTTTAGAAAAGCTCATTCATAACATAGATGTATTTACAACATTGAAGCTTGAAGTAGAGGATATTGCAAGTTACAAGAGTGCCTATGGAGTAGAGGGAAAATGGTCAATTGAGCAAATTCGTACTCTGTTAAACTACAAAACACTTAAAGCATCATATTCCACGCTACCTAAATACATTAACTGGTATGACTACAGCAGTGACAAAGTAACGGAGAAAATGGCTCAGCTCACTGGTTGGAATAAAGATACACTTGAAGCTATTAAAAAAGTTGAAGTATTTAAGCAATGCTTTGGCAAGCAAGATCCGGTGAATTCTTTGATGAGAATCAAATCTGTTATGGACATCACAGGAATTAACGTAAAAATTCTACTAAAGTTAAAGGATTTATATAATTTAAAAGCAAGTAATGGATGGAATAAATACACCGATATTGCTGGGGATTTAGAGTTAATCACCGAAAAAGATGAAGGATATTTAGCAAGAGAAAAGCGAGATATCCTAGCAAGGTACATGATACATATCAATCCAGACTTAAAAACCATGAGGGATCTCTATGGATTCTTGCTGATAGATGTTGAAATGAGTGAATGTTCAAAGATTTCGCCGATTAAAGCAGCACTAAATAGTGTTCAGCTATACATTCATCGTGCTATGATGAAGATAGAAGAAGGTGTAGAGGTTGATAAAGACTTTACTGAAGAAAAATGGAAATGGCTATCTAGTTATCGAGAATGG

>Phage wAsoc tail region Sanger

ATGTTACCAAAGATCCTAAAGAATTTTAACGTATTCGTTGATGGTCGAGGTTATGCTGGGAAAATAGATGAAATAACTCTACCGAAGCTCACTATCAAAACAGAAGAATACCGTGCAGGGGGTATGGACATACCAATTAGCATTGATATGGGCATGGAGAAGCTTGAAGCTGATTTTACTTTTGCTGAATATGATTCAGAGCTGTTTAGACTCTTTGGTTTAATAGATGGGAATTCGGTTTCTTTGACGCTAAGAGGAGGATTACAAGGCAGCGGAAACAATGATATTGAAGGAGTAATTATCAACCTTAGGGGAATATTTAAAGAGTTTGATTTTGGTAGCTGGAAACCGGCTGAAAAAGCCACTCTCAAATGCACTGTAGCTGCCCATTATTATAAACTTACTATAGGTGGCAATGAGCTGATAGAGATTGATGCTGAAAATATGATGAGAAAGATAAATGGTGTTGATCAAATGGCTTTGCTGCAAACGGTTTTAGGCATATGAAAAGATTTCGAAATTTTATTTTAAGGAGGCAATAATGCACACTATAACACTTAATAACCCAATTACAGTTGATGGAATTTCTGTCTCAGAATTATCGATGCGTGAACCTAAAGTAAGAGATTTACTTGCTATAGAACGCATAGAAGGCGAAGCTTTGAAAGAAGTAGCTTTAATTGCTAATCTAGCGTCTGTGCCAAAAGAAGTAGTTGAAGATTTATGTATTAAAGATTATGTAAAAATACAGAAGGTGCTAAAAGATTTTTTGTCGCCGCTGGAACAGAGAACTTAAAGTATAATGTATTACTACTCAGTTCTATTTCAGGAAGTGNAATTGAGCAGATTACTAATATGGATGTAAATGAATTTTTATCCTGGATAGAAATAAGTAAAGGAGTAGCAAAAAAATGTCAATGCTTTCAATAAAGATCGGTGCAACATTAGATGGCAGTTTTAATAGTGCAATGACAGGTAGTGCAGCAAAGCTTTCTAAGTTGGGTGATAGTATAAAGCAGCTTGATTCGTCAATGAAATCTGTTTCCAAATTTAAGCAACTAAATCATGATGCCCTTGAAGCCATGAAGAATTGGAAGGAAGCGGAGAAGAAAGCAAAAGAATCAGCTGCAGCAATTGCTAAAGAGAAGAAGGAAAAAAAAGAGCCAAGCAAAGCACTGAGAAATGAATTCGAAAAATTGAAAGCATCAGCATCAAAAGCAAAAGAGGCTTATATCAAAAAGAGAGATGCTCTTCACACATTAAATGAAGAAATCAGGAAAAGTGGAAAGGATGTCAAATCTTTAGTGAGGGATCAAACTAAACTTGGTTCATCTATTGAAGTACTTAAAGGAAAATACAGTAAATTAGGATCCGTAATACAAAAACAGCAAAATGCTTTGGCAAAAAAAGCGCATTATAGATCACAAGTAATGGAGACTATAGGACTAGGTCTTTCACTTGCAGCACCAATTAAAGTTGCGATTGACTTTGAATCTGCCATGGCTGATGTTAAGAAAGTTGTAAAGTTTCAGTATGATGAAGAAATGAACGAATTCAGTGAGAATATAAAAAAATTATCCCGTGAAATACCGCTATCAGCTGCAGAGTTAGCACAAATAGCTGCAAGTGGTGGACAACTTGGTATCGACAAAGATAAGCTACTTGAATTTACTACAACAGTAGCTAAAATGGCCACAGCATTTGACATGTCTGCCGAACAAGCTGGTGATTCCATAGCTAAACTTTCTAACGTTTATGGAATTGATGTTAGTGAAATGGAACATGTTGGTAATGTGATCAACCACTTGTCAGATAACAGTGCTGCAAAAGCAAAAGATATGGTTGAAGCTCTAGCAATAGTTGGTGGTACTGCAAAACAATTTGGTCTTGATATCAAGGAAACAAGTAGTTTAGTAAATGCCTTCGTTAGTTTAGGTAAACAACCAGCAAAAGCTGCAACTGCTATAAATGCTCTACTTAGCAAACTTCAAACTGCTGAAGGACAAGGCAAAGAATTTAAAGCAGCATTGGAATCCATAGGCATAACTGCAGAAGAGATGTCACAAAAAATCGCTCAAAATGGTCAAGAAGCATTACTCTACTTTTTTAAAACTTTAGAGAAAGTAGATAAACAAGAACGTTCACAAATCCTTCTCAATCTCTTTGGTCAAGAATATCAAGATGATATTGCATTGATAGTTGGAAGCCTAAAAAAATATGAAGATGCCATAGCTTTTGTAGCTGACAAAGAAAAGTATAAACATTCCATGCAAGAAGAATTTGACAATCGCGCAAGGACTACAGCAAATAATTTGCAGCTACTCAAAAATACAATAGCAGAACTAGGAATGAACTTAGGGTCAGTTATGCTACCTCCTTTAAATTGGATAAGTAAGATTTTGAGGTCTATATCTACAGTTATAGCTTGGTTCGCAGAGAAGTGTCCAATTTTGACTACAGGAGTTATGAGCATAATTTCAGCTCTTATTATTGGCAAGATTACAGTAGTAGGTTTTGGTTATGCATTTGCATTGGCTGGAGGTGGAATATTAACTTTTAAGGCTATTTTGCAGGGAACACTTTTGCCAGTATTAACTTCATTATCAGCTCGAGTAATTCCAGCAGTAATCATGGGACTGAAAGCTTTAACGCTAGCTGTGGCTACCAATCCTATAGGAGCTGCTATTGCTGGACTATCAGTTGGTGCAGCGCTTGTAATCGCTAATTGGCAAAAGGTGAAGGACTTTTTCTCAAGTATCTGGAAATCAATTACAAAACCTATAGAAAATTGGATTGGTGTAGGAAAATTGTTTAATGATAATCCAATAAAAGCATTAGAGAACTCAGTAGAAGCAAAAATAGGCAGCACAACTAAGATAATCAGTGAAAACAATGTTTTTAGTAAAGAAAATCCTTTATTGAATAATAATGCTTTTAGTGGAATTTCGAACAGTAGAACGAATATTGAAAGTGCTAGCGTGATTACAGGAAAAGGTTCTGCAGCAGATAATTCAGAGAAAGTTTTAAAAGACTGTGAAAAGTGTGAACAAAAAATTTTCAATCAAACTTTTACATTTAATATAAGTATTAAAGCAGAACCTAACCAAGATGTACGTAGTCTTGCTGATGCAGTAATAAAAAGAATAAGGGAAAAATCACGTGATGTTCTGTTTGATTCAATAGAGCCAATTTACTAATGCTATCACTTGGTCAACATAAGCTTTCTCCAACAAGAGTAAGGTACAGTAAAGAAAATCGTTGGAGTACTATTGAGTGCATTGGTAAAACACCTTCATTACAAAATATTGGCCAAGGTACAGAAAATATAGACTTAGAAGGAATAATTTATTTTCATAATCTCAATGATTTAAATCAATTAAAAAGTATGAAAGAAGCAGAAGAACCACATACTTTAGTAGACAGTTTAGGTAATGTTTTAGGGAGGTTTGTGATTGTACGGATAGAGAAAAAACAAACATCATTTTTTCCTTGTGGATTACCAAAGAAGGTTAAGTTTAGTTTAAGTTTGAAGAGATTTAACTAAAAATGACAACATATTACTGGAGTAAGGAAGGAGAGATGATAGATCTAATTTGCTGGAGGCATTATGGAGCAACTAGAGGAGTAGTGGAAGTAGTTTTAGAGGCTAATCCTGGTCTTGCAGAGTATAGTGGTTCTTTGCCATCTGGGTTAAGGATTAAACTGCCTTTAATACAAGAGCAATTAAAAAAATCGAAGTTAAAAGTGTGGGAATAAATGAAACCTGAATTTAGCATTGAGGGAATAAAAGATCATGTAATATCAGTGCATCTTACTGATGAATCTGGTACTATAGATGATGTTGCAGAGGTATGTATTGATTATGGTAATGAAAATGTAGAAATTCCAAATGAATTAAACATAGCACTAGGTTATAAGGAAATTGGAATCTTTCCAATGGGTATATATACAGTCAACGAAGTGACTATACAGGGTCCACCTAAAACTCTACTAATAAAAGCTCATGCAACAAATTTAAGAATATCTTTAAAGGCAAAAGTATCAAAAGAATGGCACCAAATTACCATAGAAAACTTAGTAAAAGAAATAGCCCAAAAACATGGATATGGATATAAAGTTGCTGAGGAATTTAAGAATGTATTAATACCCCACATTAATCAGGTAGATGAAAGTGATATAAGTCTGTTAACAAAGATTGCAACAGAGCGTGAAGCAATGGCAAAGTTAGCTGGTGGGTATATATTGTTTATTTCAAAAAATATGGCAAAATCAGCCACAGGAAAAGTTTTAGGAACAACAACTATTAGACCTCAAGACACAATTAATTGGAAAGTGCATTTTACCGTACGTGATAAGTATAATTCAGTAGTAGCAAAATGGCACAGCTATGAAAAGGGCGAAACTATTAAAGAAACAGTTGGTAGCGGTGAGCCAAGTTATATTATGCTGGAACTTTACTCAAATGCAGAGTCAGCACTAAGTGCAGCAAATGCCAAATTAAAACAATTGAAGCGTAACAATGAAACTTTAGATATAACTATGCCTGGTAATCCGGAGTTATTTGCAGAAGCTAAACTTAGTCTTATAGGTTTTAATCAAGCGGTAGATGGTGAATGGATAATTAATAGAGCGGAGCATACTTTAAATAGCTCAGGTTATCTTACTATATTATCAGCATCTTTAAGTAAATGAAACGATGTTAAGGAAAAATAAGTATATAGTCTATTCAATGTAAAGAAACTACTGAATATGAGTCAACTAAAATATAATGAAAAGGAAAAAATACATTTTGTATGGTTTATAATATTAATGGTATGTGTTGTTATTACATATTGCTATCAAAAATCCAAAGCTACAGATAATTATAACAAAACATTACGAGCGGCTACTAGTAATTGTAACTTAGAAATAGTAAAACTTTTAATAAAGGATATGGCGCAAAACTTGAGTGAAACAGCGTTACATTATGCGGCAAGAAAAGGATGTTTAGATATTATCAAATTTCTAATATTAGAAGAGAAAGTAAATATAAATGTAATCGACAGAAATGCCTTTAAAAGGACAGCTTTACATCATGCTGTAGGTGAAGGGCATTTAGGGATTGTAAGATTCTTATTAGAAAAAGGTGCGAATCCTAACATAAAAGATAACGACGGAAAAGGAGCTAGAAAAATGGCTGTAATGGCATCGCGACACGATAAAAATAAACCTTACAGAGAAATTATTAAGCTACTTGCAAATGCAGAGGAACAGCATAAATCAAAATAGTAGTTACCAAATTAGCTATCCATAATAAATTCACATTTTAAATCGTTATGCAAAATCAAAAAATCCCAATTACGGTAATTATCACCATATTAATCCAAACCGTAGCATTGATATGGTGGTTGTCCAAGCTAGACTCAAGAGTACAGCTTCATGATAAGCTCATTGAATATGGTTTAATGGAAAAGGTACTCATACTTGAGGAAAGGGTAAAAAATCTTTCTGAGGAATTGGATGAGTTTAAATTACACGTTCTAAGCGGAAAAATTAAATCTTAATACCTTCACCTAAATTCATAATGATTAAATACATTTTATCCGTTGATGGAGGTGGAATTAGAGGAATCATACCAGTAATGATTCTAGCAGAAATAGAAAAAAGAACAAGAAGAACTATAGCTGAAATCTTTGATCTTATGGCAGGTACTTCAACTGGTGGAATTGTTGTAGCAGGGTTATGCAAGAAAGATAATCAAGGAAATCCCCAATATTCAGCCAATGATTTGGTTGAGTTCTATCGGGAATATGGACCATATATTTTCAAGTCTTCGTTTTTTAGACAATCAATACTATCTTGGTTTAACTGTGCACAATACCCACATAAAAATATTGAATCTGTACTGGATAAATATTTTGGAGAGGATATTCTAAAAAATACATTAAGTAAGGTACTGATAACAAGTTATGATATTAACAACAACTATCCTTTCTTTTTTAAGAGCTGGAGAGAAGACAGAAATTTTATCAGGCTAAAAGATGCACTCAGAGCTGCAACGGCTGCACCTACTTATTTCATACCAAAACATCT

>Phage wAsoc head decoration protein Sanger

ATGAGTAGTATAACAGAACAAAATAACCTTGGTGATCTTCTAAAATATGAAGCATCAAATTTATATTCACGAGATCAAATAACTGTCGCCAAAGGACAGAATCTCAAGCTTGGTGCAGTAGTTGCTAAAAAGACTGAAGATGGTTTTATTAGGGTCTTAAATCTAACAGCAACCGATGGCACACAAACGGCAATAGGAGTAATTTTAAGTGATGTAAACGCAAAGGAAAATGCTAAAGGAGTAATTATTGCTCGCGGTGCAATGCTAGCAGATCATGCAGTTGTATGGCCAGCAAATATCACTGAAGAGCAGAAAAATGCAGCAATAAAGCAACTTGAAACACGAGGGATCATTATCCGTAAAGCAGTCTAAAGCTATATTAAACAATAAAAAACA

>Phage major capsid protein Sanger

ATGCAAAATCCATTTACAAATACAGCATTTAGTATGACGTCACTAACTAATGCGATGAATATATTGCCGATAAATTATGGACGGGTTGAAAATTTAAATTTATTTCCAAGTAGGTCAGTAAGATTTAGACATATTACCATAGAAGAGCAAAATGGAGTATTAAGTTTACTACCAACGCAAGTACCCGGAGCACCAGCAACAGTAGGAAAAAGAGGAAAAAGAAAGGTAAGAACATTTACGATTCCACATATTCCGCATGATGATGTAGTGTTACCAGAAGAAGTACAGGGAATAAGAGCATTTGGATCAGAAAGTGAACTTAAAGCGCTGGCAGATGTAATAACTGACCATTTGCAGCTAATGAGAAATAAACATGCAATAACGTTGGAGCATTTGCGTATGGGAGCGCTGAAAGGAATAATTCTGGACGCAGATGGCAGTGAATTGTTAAATCTGTACAACGAATTTGAAATAACACCAAAAGTAGTAAATTTTGCACTGGGAGCAGCGATAACTGATGTAAAACGTAAGTGTCTGGAAGTATTGAGGCACATAGAAGATAATCTAAGTGGTGAATATATGACAGGAATTCATGCTTTAGTAAGCCCTGAGTTTTTTGATGCACTTACTTCTCATGCTAAAGTGAAAGAAGCATATGAGAGATGGCAAGAAGGAGCAGCGCTAAGAAATGATATGAGGTCAGGATTTACGTTCTGTGGAATAACGTTTGAGGAATATAGAGGACAAGCAACTGATCCTGAAGGAACCGTGAGAAGATTTATAGAAAGGGATACGGGGCACTGTTTTCCAGTAGGAACAGC

>Phage minor capsid protein Sanger

ATGGTAGAACCAAGGAGTTTTGAGTTACTGTCACTACAAACAGGAAAGCAGCCTATCTTTAAAAATATAAAACATGCAGTAAGAAATAGTGAAAGAGGAATAATACCGATACATGGCATTTTAACTAAAAAACCTGGTGCATTTGGTGAAATGCTCGGAATGACATCATATGAGCAAATAGAAGAACAAATTACACAAGCATTAGCAGATAGTAGCATAGAGACAATTATACTGGAAATAGATAGCCCCGGAGGAGAGGTAAACGGTATATTTGACCTAGCTGACTTTATTTATGAATCAAGAGGAAAAAAGAGGATAATAGCGATAGCAAATGATGATGCATATTCTGCTGCGTACGCTATAGCTTCTAGCGCTGAAAAGATTTTTCTCACCCGCACTTCAGGAGTTGGGAGTATAGGAGTAATAGCAAGTCATATAGATCAAAGTGGATTTGATGAAAAATGTGGAATAAAATATACCACAGTGTTTGCAGGAAGTAGAAAAAATGATTTAAATCCACATGAACCAATAACTTCTGAGAGTTTAGAAAATCTAAAAAGCGAAGTGAATCGTTTATATGAAATGCTGGTTGAGCTAATAGCACGGAATAGAAACCTCTCTGTAGAGGCAATAAAAAATACTGAAGCAGGGCTTTATTTTGGTGAAAATGCAGTAGAGATAGGTCTTGCAGACGGAATTACAATTCTTTCAGAGTTTAAATATATTAATAAAAACAGGAGTATTACTATGAACGAACAAACTATAACTGACCTAGAAACTAATAATTTAACTAAGTATCGTACTGAAGTTCTTGAATTAATACGTTTATGTAATATATCAAAGATGCCAGAGAAAATAGGAGAATTTATTGAGCAGAGTGTAAGTGTTGAGCAAGCAAGGGAAGTTTTAATGGAATTACTTGCAGAAAGAACAAAGAAGACAGAGATAATAAGTACAATACCACAGAATTCAGGAGAAGAGTTGATGATGCAGGTAGCGAAAAGTCGTAGGCATTTAAAATATATAACAAAAGGAGAAAAGCAAA
